# Efficacy and safety of BCMA- or GPRC5D-directed CD3 bispecific antibodies in relapsed/refractory multiple myeloma: a systematic review and meta-analysis of prospective clinical trials and real-world studies

**DOI:** 10.3389/fimmu.2026.1811816

**Published:** 2026-05-20

**Authors:** Jiashun Li, Ainikaer Abulaiti, Deyu Li, Yan Zhao, Paerhati Wahafu, Maerdan Maimaitiming, Shi Qiu, Yuxiang Zhang, Yaqin An, Wenxing Wang, Liangquan Shi, Maihemuti Yakufu, Li Shu, Guohua Li, Zhen Liu

**Affiliations:** 1 Department of Sports Medicine, Sixth Affiliated Hospital of Xinjiang Medical University, Urumqi, China; 2 Department Laboratory, The Six Affiliated Hospital, Xinjiang Medical University, Urumqi, Xinjiang, China

**Keywords:** BCMA, bispecific antibodies, GPRC5D, meta-analysis, multiple myeloma

## Abstract

**Objective:**

BCMA- or GPRC5D-directed CD3 bispecific antibodies are important T-cell–redirecting therapies for relapsed/refractory multiple myeloma (RRMM). We evaluated their efficacy, safety, and certainty of evidence.

**Methods:**

We searched the Cochrane Library, Embase, PubMed, Scopus, and Web of Science from inception to February 5, 2026. Prospective clinical trials and real-world studies of BCMA×CD3 or GPRC5D×CD3 bispecific antibodies in RRMM with extractable efficacy and safety data were included. Main efficacy outcomes were progression-free survival (PFS), stringent complete response (sCR), complete response (CR), complete response or better (≥CR), very good partial response or better (≥VGPR), and overall response rate (ORR). Safety outcomes included cytokine release syndrome (CRS), immune effector cell-associated neurotoxicity syndrome (ICANS), infection, neutropenia, anemia, and thrombocytopenia. Single-arm proportional meta-analyses, sensitivity analyses, meta-regression, and subgroup analyses were performed using STATA/MP 18.0. Methodological quality and evidence certainty were assessed using MINORS and GRADE, respectively.

**Results:**

Fifteen studies, including 17 independently extractable cohorts and 1,900 patients, were analyzed. The pooled PFS was 8.76 months (95% CI, 6.82–11.13). The pooled rates of sCR, CR, ≥CR, ≥VGPR, and ORR were 28.7%, 18.1%, 38.2%, 51.0%, and 65.9%, respectively. The pooled incidences of CRS, ICANS, infection, grade ≥3 infection, neutropenia, and grade ≥3 neutropenia were 67.5%, 9.7%, 60.3%, 38.3%, 54.7%, and 42.6%, respectively. MINORS showed moderate-to-high methodological quality. GRADE showed low-to-very low certainty of evidence.

**Conclusion:**

BCMA- or GPRC5D-directed CD3 bispecific antibodies showed clinically meaningful antitumor activity in RRMM. However, current evidence is mainly from single-arm trials and retrospective real-world studies, and certainty of evidence remains low to very low. These findings should be interpreted cautiously, with particular attention to infection and neutropenia in clinical practice.

## 1 Introduction

Multiple myeloma (MM) is a malignant plasma cell disorder characterized by the clonal proliferation of plasma cells in the bone marrow. It is often associated with abnormal secretion of monoclonal immunoglobulins or light chains. It can lead to end-organ damage, including anemia, bone destruction, renal impairment, hypercalcemia, and immune dysfunction (1, 2). In recent years, proteasome inhibitors, immunomodulatory drugs, anti-CD38 monoclonal antibodies, and other novel agents have improved outcomes for patients with MM. However, MM remains largely incurable. Most patients eventually develop relapsed or treatment-resistant disease. Patients with relapsed/refractory multiple myeloma (RRMM), especially those who have received multiple prior lines of therapy or are triple-class exposed or triple-class refractory, have limited benefit from conventional salvage therapy. This population continues to have a major unmet clinical need (3, 4).

With improved understanding of the MM immune microenvironment, tumor-associated antigens, and T-cell–redirecting mechanisms, BCMA-directed cellular and antibody-based immunotherapies have reshaped the treatment landscape of RRMM (5, 6). Chimeric antigen receptor T-cell (CAR-T) therapy is one of the most representative T-cell–redirecting approaches in RRMM. The KarMMa study showed that idecabtagene vicleucel (ide-cel) achieved high response rates in heavily pretreated patients with RRMM. The CARTITUDE-1 study and its long-term follow-up also showed that ciltacabtagene autoleucel (cilta-cel) induced deep and durable responses (7–9). However, CAR-T therapy still has important limitations. These include a long manufacturing time, the need for leukapheresis and lymphodepleting chemotherapy, limited accessibility, the need for bridging therapy in patients with rapidly progressive disease, and toxicity concerns such as cytokine release syndrome (CRS), immune effector cell-associated neurotoxicity syndrome (ICANS), prolonged cytopenias, and infections (10, 11). Therefore, there remains a need for immune therapies that can be started more rapidly, are more accessible, and can be delivered in a standardized manner.

Bispecific antibodies (BsAbs) have emerged as an important class of T-cell–engaging immunotherapies in hematologic malignancies (12). These agents bind both a tumor-associated antigen and CD3 on T cells. This dual binding redirects T cells toward myeloma cells and induces tumor cell killing. Unlike individualized CAR-T therapy, BsAbs are off-the-shelf agents. They usually do not require ex vivo cell collection or manufacturing. They can therefore be initiated more rapidly and may be clinically useful for patients who cannot wait for CAR-T manufacturing, are not suitable for cellular therapy procedures, or relapse after CAR-T therapy (11, 13). Thus, BsAbs should not be viewed simply as replacements for CAR-T therapy. Instead, they provide an important complementary T-cell–redirecting strategy with different accessibility, administration patterns, and toxicity profiles. Several BCMA×CD3 bispecific antibodies, including teclistamab, elranatamab, linvoseltamab, and alnuctamab, and GPRC5D×CD3 bispecific antibodies, such as talquetamab, have shown high response rates in patients with RRMM (14–18). BCMA is an important target on plasma cells and myeloma cells. GPRC5D is a novel target that is highly expressed on myeloma cells and is relatively independent of BCMA expression. GPRC5D-directed therapy may therefore provide another treatment option for patients who relapse after BCMA-directed therapy (5, 6).

However, treatment-related adverse events remain important concerns with BsAbs. These include CRS, ICANS, infections, neutropenia, anemia, and thrombocytopenia (16–19). In addition, included studies differ in drug target, drug family, study design, trial phase, prior BCMA-directed therapy, and prior T-cell–redirecting therapy exposure. These factors may affect pooled estimates of efficacy and safety outcomes. Therefore, this systematic review and meta-analysis aimed to evaluate the efficacy and safety of BCMA×CD3 and GPRC5D×CD3 bispecific antibodies in patients with RRMM. We also discuss their potential clinical role in the RRMM treatment sequence in the context of CAR-T therapy.

## 2 Methods

Declaration: This systematic review and meta-analysis was conducted and reported in accordance with the PRISMA 2020 statement. No prospective protocol registration was completed before study screening and data extraction.

### 2.1 Search strategy

We systematically searched the Cochrane Library, Embase, PubMed, Scopus, and Web of Science from database inception to February 5, 2026. The search strategy included MeSH terms and free-text keywords related to “multiple myeloma” and “bispecific antibodies.” Terms within each concept were combined with the OR operator, and the two main concepts were combined with the AND operator. The complete search strategy and detailed search terms are provided in **Supplementary File 1**. To ensure comprehensive coverage, the search included grey literature, such as conference abstracts and original research articles, without any restrictions on publication type.

### 2.2 Inclusion and exclusion criteria

#### 2.2.1 Inclusion criteria

This review included both approved and investigational BCMA×CD3 or GPRC5D×CD3 bispecific antibodies when extractable clinical efficacy and safety data were available from prospective clinical studies or real-world cohorts.

Studies were eligible if they met the following criteria: (1) the study population consisted of patients with RRMM who received BCMA×CD3 or GPRC5D×CD3 bispecific antibody therapy; (2) the follow-up duration was at least 3 months, or, when median follow-up was not reported, all included patients had completed at least one protocol-defined response assessment and a clear data cutoff date was available; (3) at least one prespecified efficacy outcome was reported, including ORR, ≥VGPR, ≥CR, CR, sCR, or PFS; (4) at least one prespecified safety outcome was reported, including CRS, ICANS, infection, neutropenia, anemia, or thrombocytopenia; (5) extractable numerator and denominator data were available, or event counts could be reconstructed from reported percentages and sample sizes; and (6) for multiple reports from the same study, we prioritized peer-reviewed full-text publications, the largest eligible sample size, the longest follow-up, and data from the approved dose or recommended phase 2 dose cohort.

#### 2.2.2 Exclusion criteria

Studies were excluded if they met any of the following criteria: (1) the intervention was not a BCMA×CD3 or GPRC5D×CD3 bispecific antibody, including antibody-drug conjugates, CAR-T-cell therapies, monoclonal antibodies, or other non-BsAb therapies; (2) the article was a case report, review, commentary, basic science study, or animal study; (3) prespecified efficacy or safety outcome data could not be extracted; (4) the report was a duplicate or suspected duplicate publication; (5) multiple overlapping subgroup reports were derived from the same real-world parent cohort, in which case only the largest, non-overlapping, and most representative dataset was retained for the main meta-analysis; or (6) the report was a conference abstract with a corresponding peer-reviewed full-text publication or without sufficient extractable data. Conference abstracts were included only when no corresponding full-text publication was available and the required eligibility data could be extracted.

### 2.2.3 Data extraction and quality assessment

Two reviewers independently screened the records and extracted the required data. Disagreements were resolved through discussion with a third reviewer. The extracted information included the first author, publication year, clinical trial registration number when available, study phase, sample size, prior therapies, treatment received during the study, sex distribution, age, proportion of patients with high-risk cytogenetic abnormalities (HRCA), follow-up duration, and efficacy and safety outcomes.

The certainty of evidence was assessed using the Grading of Recommendations Assessment, Development and Evaluation (GRADE) framework. Because the included evidence was mainly derived from single-arm prospective clinical trials and retrospective observational studies, the initial certainty of evidence was rated as low. Two reviewers independently assessed the body of evidence for each major outcome, including ORR, ≥VGPR, ≥CR, CR, sCR, PFS, CRS, ICANS, infection, neutropenia, grade ≥3 infection, and grade ≥3 neutropenia. CRS and ICANS were extracted according to the grading criteria reported in each original study. When applicable, these events were interpreted according to the ASTCT consensus grading framework for immune effector cell-associated toxicities (20). High-risk cytogenetic abnormalities were extracted as defined in the original studies and interpreted according to contemporary IMWG/IMS criteria when applicable (21).

The assessment considered five domains: study limitations or risk of bias, inconsistency, indirectness, imprecision, and publication bias. The final certainty of evidence was categorized as high, moderate, low, or very low. Disagreements were resolved through discussion with a third reviewer.

The methodological quality of the included studies was assessed using the Methodological Index for Non-Randomized Studies (MINORS) for non-comparative studies (22). Each item was scored as 0, 1, or 2, corresponding to not reported, reported but inadequate, or adequately reported, respectively. The maximum score for non-comparative studies was 16. The eight assessed items included a clearly stated aim, inclusion of consecutive patients, prospective collection of data, endpoints appropriate to the study aim, unbiased assessment of endpoints, follow-up period appropriate to the study aim, loss to follow-up of less than 5%, and prospective calculation of study size. Two reviewers independently performed the assessment, and disagreements were resolved through discussion with a third reviewer. For studies with multiple independently extractable cohorts from the same report, MINORS was assessed at the study or report level rather than repeatedly at the cohort level.

### 2.2.4 Statistical analysis

All statistical analyses were performed using STATA/MP 18.0. For single-arm proportional outcomes, pooled estimates were calculated after logit transformation. The pooled proportions and 95% confidence intervals (CIs) were then presented after back-transformation. Heterogeneity was assessed using the I² statistic and Cochran’s Q test. A random-effects model was used when I² was greater than 50%; otherwise, a fixed-effect model was applied. Leave-one-out sensitivity analysis, univariable meta-regression, and subgroup analyses were further performed for prespecified major outcomes. Publication bias was primarily assessed using funnel plots. For major outcomes with a sufficient number of studies, Egger’s test and Begg’s test were also used to assess small-study effects. A two-sided P value <0.05 was considered statistically significant.

## 3 Results

### 3.1 Study characteristics

**Figure 1:**
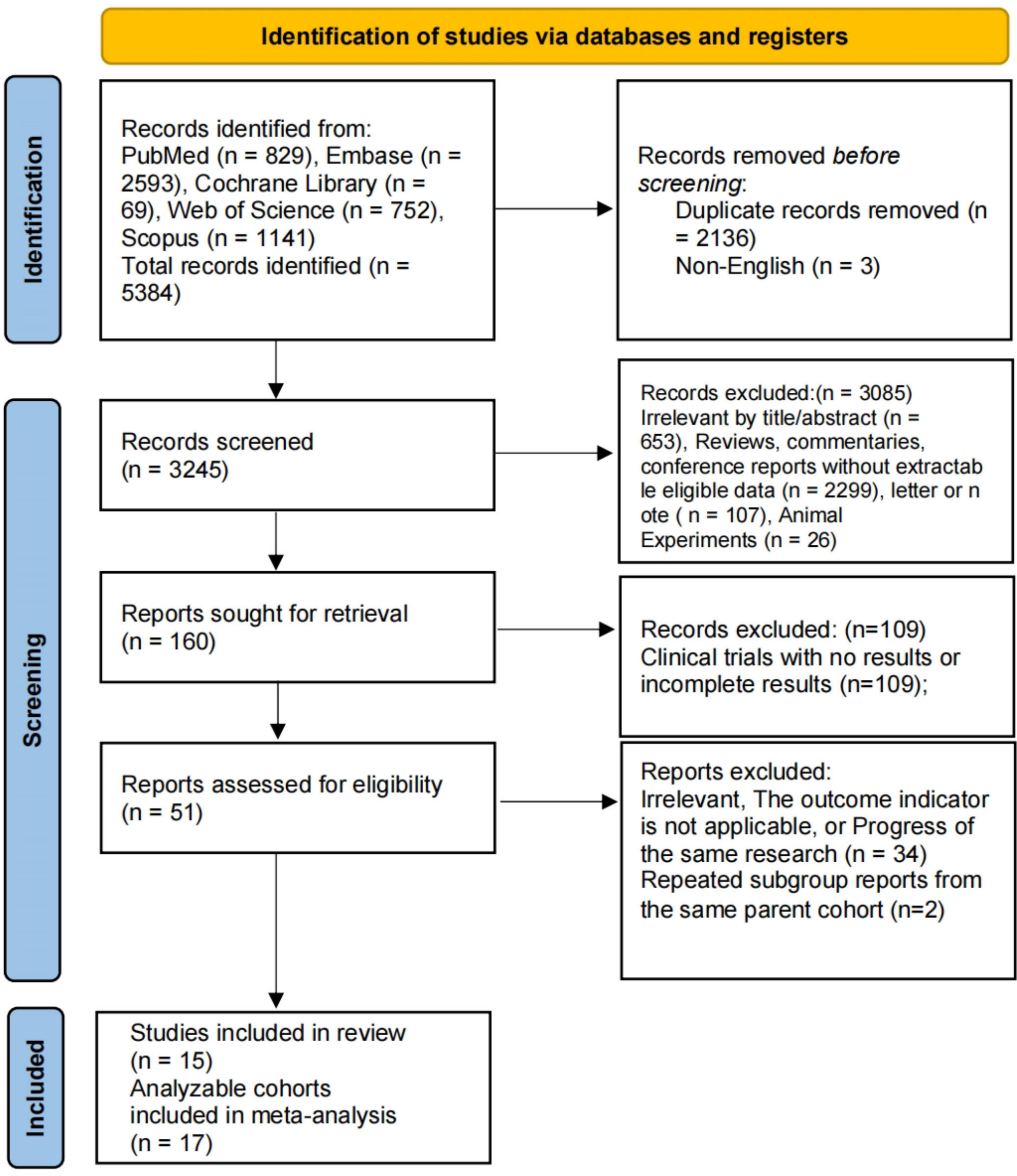
Screening process.

The initial search identified 5,384 records. After screening, 15 original studies, comprising 17 independently extractable analytic cohorts, were included. These studies involved 1,900 patients with RRMM. The study selection process is shown in **Figure 1**. By treatment target, 1,289 patients received BCMA×CD3 bispecific antibodies, and 611 patients received GPRC5D×CD3 bispecific antibodies. The included studies consisted of 10 prospective clinical trials or trial-extension cohorts and 5 retrospective real-world studies. The evaluated agents included talquetamab, elranatamab, alnuctamab, linvoseltamab, ABBV-383, and teclistamab. The baseline characteristics of the included studies are provided in **Supplementary File 2**. Extractable efficacy and safety outcomes are summarized in **Tables 1**, **2**, respectively. Because the number of contributing studies and evaluable patients varied across outcomes, each pooled analysis was performed using the available numerator and denominator data for that specific outcome.

**Table 1:** Efficacy outcomes.

Study	Median DOR (months)	Median PFS (months)	Median OS (months)	sCR	CR	≥CR	≥VGPR	ORR
Al Hadidi.2025 (23)	NR	10	NR	NR	25/98	NR	25/98	72/98
An.2025A (24)	15.7 (5.7-NE)	8.3 (6.3-NE)	NR (14.5-NE)	NR	NR	11/29	17/29	20/29
An.2025B (24)	NR (2.8-NE)	NR (2.3-NE)	NR	NR	NR	6/12	7/12	8/12
Bahlis.2023 (25)	17.1 (11.1-NE)	11.8 (6-19.1)	21.2 (10.9-NA)	15/55	6/55	21/55	31/55	35/55
Bar.2026 (26)	NR	NR	NR	12/49	6/49	18/49	26/49	35/49
Bumma.2024 (27)	29.4 (19.2-NE)	NR	31.4 (21.6-NE)	NR	NR	58/117	74/117	83/117
Chari.2025A (16)	9.5 (6.7-13.4)	7.5 (5.7-9.4)	NR	NR	NR	NR	NR	106/143
Chari.2025B (16)	16.9 (12.9-NE)	11.2 (8.4-16.9)	NR	NR	NR	NR	NR	107/154
D’Souza.2022 (28)	NR	NR	NR	16/79	13/79	29/79	43/79	54/79
Frenking.2025 (29)	12.7 (9.5-NE)	6.4 (5.1-9.0)	NR	NR	NR	NR	32/123	80/123
Lesokhin.2023 (15)	NR	NR	NR	NR	NR	43/123	69/123	75/123
Mohan.2024 (30)	NR	NR	NR	NR	19/98	NR	49/98	61/98
Moreau.2022 (14)	18.4 (14.9-NE)	11.3 (8.8-17.1)	18.3 (15.1-NE)	NR	NR	65/165	97/165	104/165
Razzo.2025 (31)	NR	5.8	NR	NR	NR	130/506	228/506	270/506
Shigeki.2025 (32)	NR (10.2-NE)	15.5 (11.3-NE)	NR (15.2-NE)	17/36	3/36	20/36	26/36	28/36
Touzeau.2024 (33)	14.8 (8.0-22.6)	4.5 (1.3-11.6)	15.5 (8.3-27.9)	NR	NR	12/40	19/40	21/40
Yi.2025 (34)	NR	14.1	NR	NR	NR	17/42	22/42	28/42

DOR, Duration of Response; PFS, Progression-Free Survival; OS, Overall Survival; sCR, stringent complete response; CR, Complete Response; VGPR, Very good partial response; ORR, Overall response rate, defined as partial response or better.

**Table 2:** Safety outcomes.

Study	CRS	ICANS	Neutropenia	Grade ≥3 neutropenia	Infection	Grade ≥3 infection	Grade ≥3 anemia	Grade ≥3 thrombocytopenia
Al Hadidi.2025 (23)	62/114	11/114	NR	NR	31/114	21/114	NR	NR
An.2025A (24)	26/29	1/29	21/29	9/29	23/29	15/29	8/29	5/29
An.2025B (24)	10/12	0/12	8/12	2/12	5/12	2/12	3/12	1/12
Bahlis.2023 (25)	48/55	9/55	41/55	39/55	41/55	15/55	28/55	16/55
Bar.2026 (26)	28/49	NR	23/49	20/49	34/49	5/49	8/49	6/49
Bumma.2024 (27)	54/117	9/117	50/117	49/117	87/117	42/117	36/117	NR
Chari.2025A (16)	113/143	13/122	50/143	44/143	85/143	29/143	45/143	29/143
Chari.2025B (16)	115/154	12/118	44/154	33/154	105/154	28/154	40/154	28/154
D’Souza.2022 (28)	58/79	/	36/79	32/79	/	/	13/79	9/79
Frenking.2025 (29)	86/123	11/123	73/123	32/123	75/123	34/123	/	/
Lesokhin.2023 (15)	86/123	4/123	60/123	60/123	86/123	49/123	46/123	29/123
Mohan.2024 (30)	62/110	12/110	/	/	44/110	29/110	/	/
Moreau.2022 (14)	119/165	5/165	117/165	106/165 *	126/165	74/165	61/165*	35/165 *
Razzo.2025 (31)	276/509	57/509	/	/	214/509	/	/	/
Shigeki.2025 (32)	27/36	0/36	14/36	10/36	19/36	6/36	8/36	
Touzeau.2024 (33)	26/40	4/40	28/40	26/40	28/40	13/40	14/40	12/40
Yi.2025 (34)	20/42	/	31/42	29/42	20/42	18/42	14/42	21/42

CRS, Cytokine release syndrome; ICANS, immune effector cell-associated neurotoxicity syndrome. *Event counts marked with an asterisk were reconstructed from the reported percentages and corresponding sample sizes in the original articles; Denominators may differ across outcomes because some studies reported outcome-specific assessable populations.

The GRADE assessment showed that the certainty of evidence for the major efficacy and safety outcomes ranged from low to very low. The certainty of evidence was rated as low for CR, ORR, and ICANS. This was mainly because these outcomes showed relatively stable pooled estimates, low or explainable heterogeneity, and no clear evidence of publication bias. The certainty of evidence was rated as very low for PFS, sCR, ≥CR, ≥VGPR, CRS, neutropenia, grade ≥3 neutropenia, infection, grade ≥3 infection, grade ≥3 anemia, and grade ≥3 thrombocytopenia. The main reasons for downgrading were the absence of randomized controlled studies, substantial between-study heterogeneity for some outcomes, and differences across studies in drug target, drug family, study design, trial phase, and prior exposure to BCMA-directed or T-cell–redirecting therapies. Publication bias was assessed for all prespecified outcomes and was not considered a major reason for downgrading.

The MINORS assessment showed that the overall methodological quality of the included studies was moderate to high. The total MINORS scores of the 15 studies ranged from 11 to 14. Prospective clinical trials or trial-extension cohorts generally received higher scores. These studies commonly had clearly stated aims, appropriate endpoints, adequate follow-up, and extractable outcome data. Retrospective real-world studies were generally rated as moderate quality. The main reasons for score deduction were the lack of prospective data collection, incomplete reporting of consecutive patient inclusion or loss to follow-up, and the absence of prospective sample size calculation in most studies. Overall, no included study was rated as low quality. However, because most studies were single-arm, open-label, or retrospective, potential selection bias and information bias could not be fully excluded. The full GRADE evidence profile and MINORS assessment are provided in **Supplementary File 3**.

### 3.3 Additional analytical strategy for meta-regression and subgroup analysis

Because this review included multiple outcomes, and because the number of contributing studies and the sparsity of events differed across outcomes, meta-regression was performed only for prespecified major efficacy and safety endpoints. These included ORR, deep response outcomes (≥VGPR and ≥CR), CRS, infection, and grade ≥3 infection. For secondary outcomes with few contributing studies, sparse events, or inconsistent definitions, only overall meta-analysis and sensitivity analysis were performed. Formal meta-regression was not conducted for these outcomes to avoid model instability and redundant results.

Univariable random-effects meta-regression was performed only for prespecified core efficacy and safety outcomes. Other secondary outcomes were analyzed using overall pooling and sensitivity analyses because of limited study numbers, sparse events, or clinically heterogeneous definitions.

Subgroup analyses were performed according to the following hierarchy: first, drug target: [(1). BCMA×CD3 vs (2). GPRC5D×CD3]; second, study method: [(1). prospective trial vs (2). retrospective real-world study]; third, drug family [(1). teclistamab, (2). talquetamab, or (3). other BCMA-directed bispecific antibodies)]; fourth, prior targeted or T-cell–redirecting therapy exposure: [(1). naive/restricted exposure, (2). mixed exposure, or (3). required prior BCMA exposure]; and fifth, trial phase or evidence level: [(1). phase I, (2). phase I/II or phase II registrational study, or (3). real-world post-approval study].

### 3.4 Efficacy outcomes

**Figure 2:**
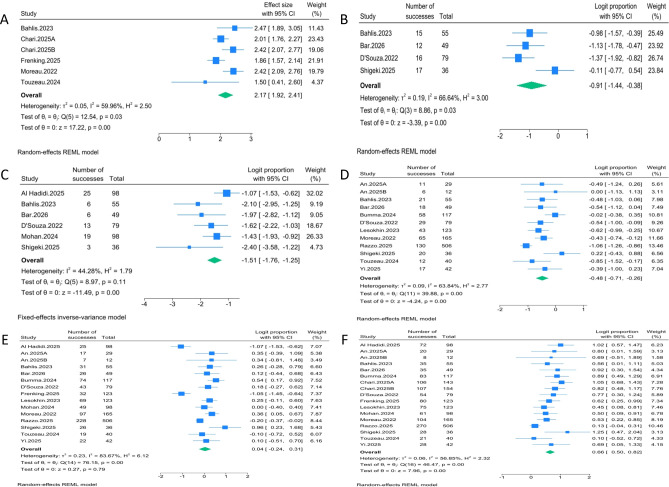
Meta-analysis of the efficacy after bispecific antibody treatment: **(A)** PFS, **(B)** sCR, **(C)** CR, **(D)** ≥CR, **(E)** ≥VGPR, **(F)** ORR.

We analyzed the following efficacy outcomes: progression-free survival (PFS), stringent complete response (sCR), complete response (CR), complete response or better (≥CR), very good partial response or better (≥VGPR), and overall response rate (ORR). Six cohorts reported PFS. The pooled median PFS was 8.76 months (95% CI, 6.82–11.13), with moderate heterogeneity (I² = 59.96%). Four cohorts reported sCR, with a pooled sCR rate of 28.7% (95% CI, 19.2%–40.6%; I² = 66.64%). Six cohorts reported CR, with a pooled CR rate of 18.1% (95% CI, 14.7%–22.3%; I² = 44.28%). Twelve cohorts reported ≥CR, with a pooled ≥CR rate of 38.2% (95% CI, 33.0%–43.5%; I² = 63.84%). Fifteen cohorts reported ≥VGPR, with a pooled ≥VGPR rate of 51.0% (95% CI, 44.0%–57.7%; I² = 83.67%). Seventeen cohorts reported ORR, with a pooled ORR of 65.9% (95% CI, 62.2%–69.4%; I² = 56.85%). Forest plots for efficacy outcomes are shown in **Figure 2**.

### 3.5 Safety outcomes

**Figure 3:**
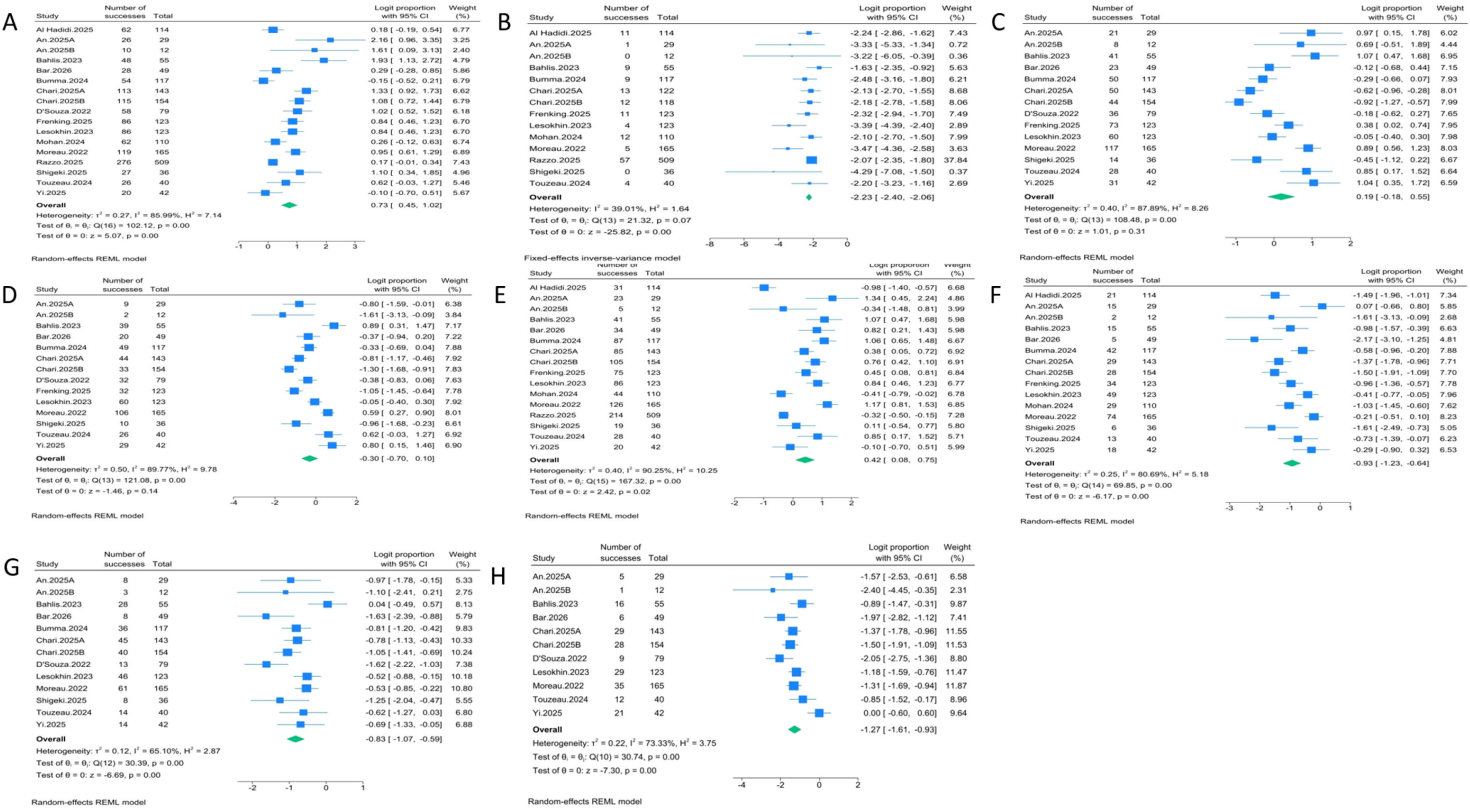
Meta-analysis of Safety Outcomes after bispecific antibody therapy. **(A)** CRS, **(B)** ICANS, **(C)** Neutropenia, **(D)** Grade ≥3 Neutropenia, **(E)** Infection, **(F)** Grade ≥3 Infection, **(G)** Grade ≥3 Anemia, **(H)** Grade ≥3 Thrombocytopenia.

We analyzed the following safety outcomes: CRS, ICANS, neutropenia, grade ≥3 neutropenia, infection, grade ≥3 infection, grade ≥3 anemia, and grade ≥3 thrombocytopenia. Seventeen cohorts reported CRS, with a pooled incidence of 67.5% (95% CI, 61.1%–73.5%; I² = 85.99%). Fourteen cohorts reported ICANS, with a pooled incidence of 9.7% (95% CI, 8.3%–11.3%; I² = 39.01%). Fourteen cohorts reported neutropenia and grade ≥3 neutropenia. The pooled incidences were 54.7% (95% CI, 45.5%–63.4%; I² = 87.89%) and 42.6% (95% CI, 33.2%–52.5%; I² = 89.77%), respectively. Sixteen cohorts reported infection, and 15 cohorts reported grade ≥3 infection. The pooled incidences were 60.3% (95% CI, 52.0%–67.9%; I² = 90.25%) and 38.3% (95% CI, 31.6%–44.9%; I² = 63.84%), respectively. Thirteen cohorts reported grade ≥3 anemia, with a pooled incidence of 30.4% (95% CI, 25.5%–35.7%; I² = 65.10%). Seven cohorts reported grade ≥3 thrombocytopenia, with a pooled incidence of 21.9% (95% CI, 16.3%–28.3%; I² = 73.33%). Forest plots for safety outcomes are shown in **Figure 3**. The efficacy and safety outcomes analyses are summarized in **Table 3**.

**Table 3:** Meta-analysis summary.

Outcome effect	Cohorts, n	Pooled estimate	95% CI	I² (%)	Model
Efficacy outcomes					
PFS, months	6	8.76	6.82–11.13	59.96	Random-effects
sCR	4	28.7%	19.2%–40.6%	66.64	Random-effects
CR	6	18.1%	14.7%–22.3%	44.28	Fixed-effect
≥CR	12	38.2%	33.0%–43.5%	63.84	Random-effects
≥VGPR	15	51.0%	44.0%–57.7%	83.67	Random-effects
ORR	17	65.9%	62.2%–69.4%	56.85	Random-effects
Safety outcomes					
CRS	17	67.5%	61.1%–73.5%	85.99	Random-effects
ICANS	14	9.7%	8.3%–11.3%	39.01	Fixed-effect
Neutropenia	14	54.7%	45.5%–63.4%	87.89	Random-effects
Grade ≥3 neutropenia	14	42.6%	33.2%–52.5%	89.77	Random-effects
Infection	16	60.3%	52.0%–67.9%	90.25	Random-effects
Grade ≥3 infection	15	38.3%	31.6%–44.9%	63.84	Random-effects
Grade ≥3 anemia	13	30.4%	25.5%–35.7%	65.10	Random-effects
Grade ≥3 thrombocytopenia	7	21.9%	16.3%–28.3%	73.33	Random-effects

CI, confidence interval; PFS, progression-free survival; sCR, stringent complete response; CR, complete response; VGPR, very good partial response; ORR, overall response rate; CRS, cytokine release syndrome; ICANS, immune effector cell-associated neurotoxicity syndrome.

### 3.6 Publication bias assessment

**Figure 4:**
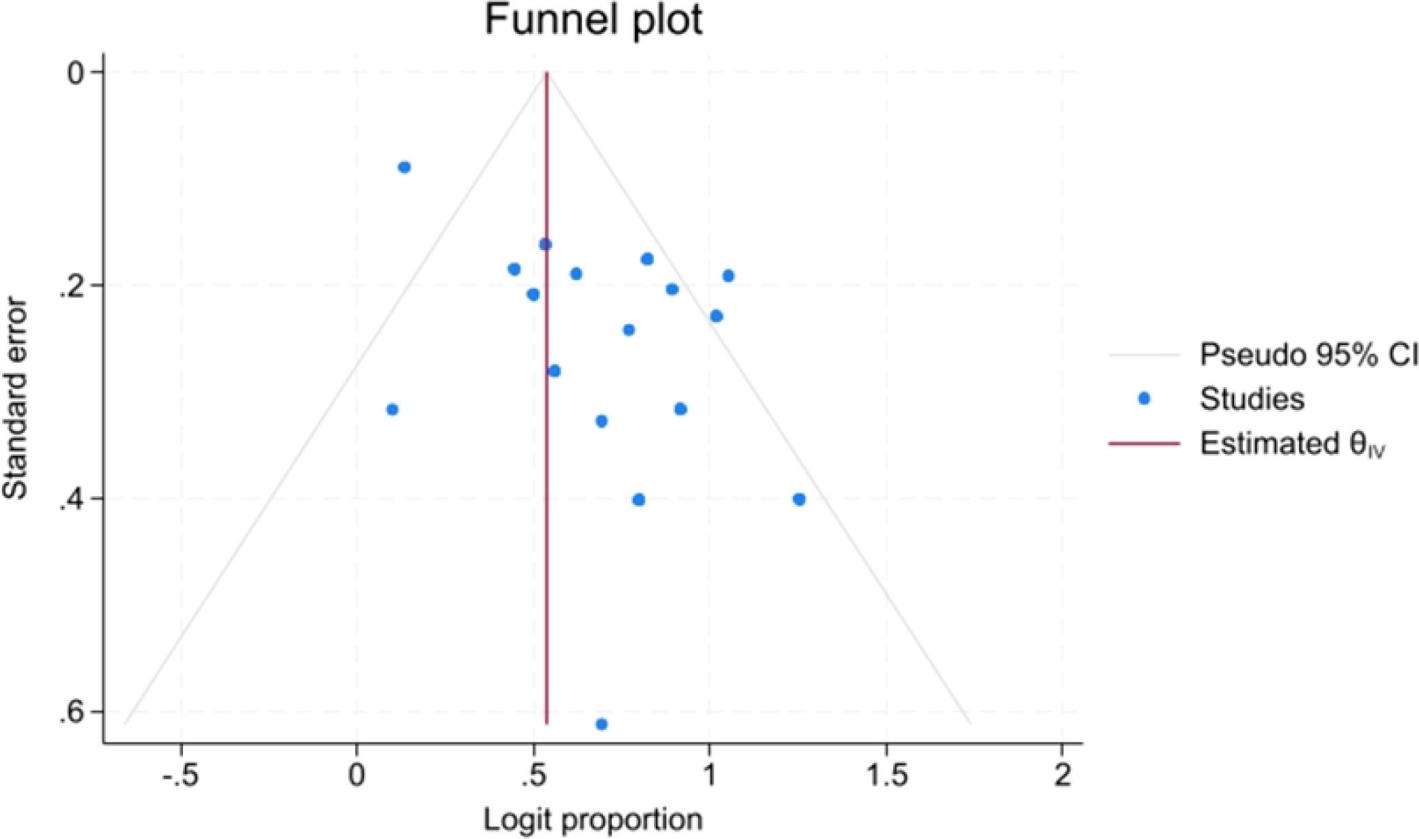
Funnel plot for the ORR outcome.

Publication bias and small-study effects were formally assessed first for ORR, which was the clinically most important efficacy endpoint and had a sufficient number of contributing cohorts. Visual inspection of the ORR funnel plot showed no obvious asymmetry (**Figure 4**). Egger’s test (P = 0.1213) and Begg’s test (P = 0.5366) did not suggest significant small-study effects. Funnel plots were also generated for the remaining efficacy and safety outcomes as supplementary assessments. Overall, no clear evidence of publication bias was observed. However, several outcomes included a limited number of cohorts, and funnel plots in single-arm proportion meta-analyses can be affected by between-study heterogeneity and sample-size distribution. Therefore, publication bias assessments for outcomes other than ORR should be interpreted with caution. Funnel plots for all outcomes are provided in **Supplementary File 8**.

### 3.7 Sensitivity analysis, meta-regression, and subgroup analysis

#### 3.7.1 Sensitivity analysis

For sCR, the leave-one-out sensitivity analysis showed that sequential omission of any single study did not change the direction of the pooled effect. This suggests that the overall result was relatively robust. Shigeki.2025et al. (32) had a relatively large influence on the pooled estimate and heterogeneity. After excluding this study, heterogeneity decreased to 0%. However, the direction of the pooled effect and the statistical conclusion did not change materially. This indicates that Shigeki.2025 mainly contributed to between-study heterogeneity but did not alter the overall conclusion.

For the remaining outcomes, leave-one-out sensitivity analyses showed only small changes in the pooled estimates after sequential omission of each study. No single study had a decisive influence on the overall results. These findings suggest that the pooled estimates were generally stable. The full sensitivity analyses for all outcomes are provided in **Supplementary File 4**.

#### 3.7.2 Meta-regression

For efficacy outcomes, meta-regression suggested that the potential sources of heterogeneity differed across outcomes. For ORR, drug target and prior treatment background were statistically associated with the pooled effect. For ≥VGPR, study design, prior treatment background, and trial phase may have been associated with the pooled effect. For ≥CR, study design and prior treatment background were statistically associated with the pooled effect. For CR, study design and trial phase may have been associated with the pooled effect.

For safety outcomes, meta-regression also suggested that study-related factors may have influenced the pooled estimates of adverse events. For CRS, study design and trial phase were statistically associated with the pooled effect. For ICANS, study design and trial phase may have been associated with the pooled effect. For neutropenia, prior treatment background was statistically associated with the pooled effect. For grade ≥3 neutropenia, drug target was significantly associated with the pooled effect. For infection, study design and trial phase were significantly associated with the pooled effect. For grade ≥3 infection, study design and prior treatment background may have been associated with the pooled effect.

It should be noted that meta-regression was based on study-level covariates, and some outcomes included a limited number of studies. Therefore, these findings should be considered exploratory and should not be interpreted as causal. The full meta-regression results and covariate-specific outputs are provided in **Supplementary File 5**.

#### 3.7.3 Subgroup analysis

Exploratory subgroup analyses were performed for selected major outcomes based on clinical relevance, data availability, and potential sources of heterogeneity suggested by meta-regression.

##### 3.7.3.1 Efficacy outcomes

For CR, subgroup analysis by trial phase showed significant between-subgroup differences. The pooled CR rates in the three trial-phase subgroups were 13.8%, 8.3%, and 22.6%, respectively (P = 0.03). When stratified by study design, the pooled CR rates were 13.1% and 22.6% in the two subgroups, respectively, and the between-subgroup difference was also significant (P = 0.01). These findings suggest that trial phase and study design may influence the pooled CR rate. However, the result should be interpreted with caution because the second trial-phase subgroup, defined as phase I/II or phase II registrational studies, included only the study by Shigeki et al. (32).

For ≥CR, although study design and prior treatment background may have been associated with the pooled effect, subgroup analysis did not show significant between-subgroup differences. For ≥VGPR, a significant difference was observed between prospective clinical trial cohorts and retrospective real-world cohorts (P < 0.001). The pooled estimate was higher in prospective trial cohorts, with low within-subgroup heterogeneity (I² = 0.00%). In contrast, the pooled estimate was lower in real-world cohorts, with substantial heterogeneity (I² = 88.59%). This suggests that study design may be an important source of heterogeneity for ≥VGPR. Exploratory subgroup analyses by prior treatment background and trial phase also showed significant or trend-level between-subgroup differences for ≥VGPR.

For ORR, subgroup analysis showed a significant difference between BCMA×CD3 and GPRC5D×CD3 bispecific antibodies (P = 0.01). The pooled estimate was higher in the GPRC5D×CD3 subgroup than in the BCMA×CD3 subgroup, and within-subgroup heterogeneity was low (I² = 0%). This suggests that drug target may be an important source of heterogeneity for ORR. Exploratory subgroup analyses by drug family and prior treatment background also showed significant or trend-level differences. Subgroup analyses for efficacy outcomes are provided in **Supplementary File 6**.

##### 3.7.3.2 Safety outcomes: CRS, ICANS, and neutropenia

For CRS, significant between-subgroup differences were observed by study design and trial phase. However, heterogeneity remained high within several subgroups. For ICANS, subgroup analysis by prior treatment exposure showed a significant between-subgroup difference (P = 0.04), suggesting that prior treatment background may be a potential source of variation in ICANS incidence. This result should be interpreted cautiously because the third subgroup included only Touzeau.2024 (33).

For neutropenia, significant between-subgroup differences were observed across prior treatment exposure groups. This suggests that neutropenia may occur more frequently in cohorts with more complex prior treatment exposure. However, within-subgroup heterogeneity remained high. For grade ≥3 neutropenia, subgroup analysis suggested that prior treatment exposure may influence its incidence, with significant differences across prior treatment background subgroups. When stratified by drug target, the pooled incidence was approximately 54.7% (95% CI, 45.5%–63.9%) in the BCMA×CD3 subgroup and 26.3% (95% CI, 22.3%–31.0%) in the GPRC5D×CD3 subgroup. The between-subgroup difference was statistically significant (P < 0.001). These findings suggest that drug target and prior treatment background may be major sources of heterogeneity for grade ≥3 neutropenia.

##### 3.7.3.3 Safety outcomes: infection and grade ≥3 infection

For infection, subgroup analysis by study design showed a significant difference between prospective trial cohorts and retrospective real-world cohorts. The incidence of infection was higher in prospective trial cohorts than in real-world cohorts, suggesting that study design may be an important source of heterogeneity for infection. Significant differences were also observed when studies were stratified by trial phase. However, because trial phase and study design partly overlap, this finding should be interpreted as supportive evidence.

Overall, between-study variation in infection outcomes may mainly reflect differences in study design and evidence level. For grade ≥3 infection, study design and prior treatment background may have been associated with the pooled effect, but subsequent subgroup analyses did not show significant between-subgroup differences. Subgroup analyses for safety outcomes are provided in **Supplementary File 7**.

## 4 Discussion

This systematic review and meta-analysis included 15 studies and 17 analytic cohorts, comprising 1,900 patients with RRMM. We comprehensively evaluated the efficacy and safety of BCMA×CD3 and GPRC5D×CD3 bispecific antibodies. Overall, BsAbs showed substantial antitumor activity in heavily pretreated patients with RRMM. The pooled ORR was 65.9%, the ≥VGPR rate was 51.0%, the ≥CR rate was 38.2%, the CR rate was 18.1%, the sCR rate was 28.7%, and the pooled median PFS was 8.76 months. For safety outcomes, CRS, infection, and hematologic toxicities were the most common adverse events. The pooled incidences of CRS, infection, grade ≥3 infection, and grade ≥3 neutropenia were 67.5%, 60.3%, 38.3%, and 42.6%, respectively. These findings suggest that BsAbs can induce clinically meaningful responses in RRMM. However, infection and myelosuppression remain important safety concerns in clinical practice.

From an efficacy perspective, the pooled ORR was close to two-thirds. This finding suggests that BCMA×CD3 and GPRC5D×CD3 bispecific antibodies have consistent antimyeloma activity in heavily pretreated patients with RRMM, as also reported in pivotal clinical trials of teclistamab, elranatamab, and talquetamab (14–16). Real-world studies of teclistamab and talquetamab further support the clinical activity of these agents outside trial settings (23, 29–31). The ≥VGPR and ≥CR results indicate that BsAbs can induce not only partial responses but also deep responses in a substantial proportion of patients. However, response depth varied across outcomes. The sCR, CR, and ≥CR rates were lower than the ORR and ≥VGPR rates. This may be related to differences in baseline disease burden, prior treatment exposure, completeness of bone marrow assessment, minimal residual disease testing, and follow-up duration. According to International Myeloma Working Group response criteria, confirmation of CR and sCR requires more stringent assessment than ORR or VGPR (35). In real-world cohorts, some patients may not undergo complete bone marrow confirmation or MRD testing because of disease progression, poor performance status, or routine-care limitations, which may affect the classification of deep responses (30, 31, 34).

In the current RRMM immunotherapy landscape, CAR-T-cell therapy provides an important context for interpreting the clinical role of BsAbs. The KarMMa study showed that ide-cel had clear activity in heavily pretreated patients with RRMM. The CARTITUDE-1 study and its long-term follow-up showed that cilta-cel achieved very high ORR and sCR rates and durable disease control (7–9). Compared with these CAR-T studies, the pooled outcomes in our BsAb meta-analysis showed an ORR of 65.9%, a ≥CR rate of 38.2%, and a PFS of 8.76 months. These results appear broadly comparable to the ide-cel study background but are lower than the depth of response and long-term disease control reported with cilta-cel in CARTITUDE-1. This comparison should be interpreted with caution. It is an indirect contextual comparison, not a head-to-head comparison. CAR-T and BsAb studies differ in eligibility criteria, number of prior lines of therapy, prior BCMA exposure, allowance of prior T-cell–redirecting therapy, follow-up duration, endpoint assessment, and the proportion of real-world patients. Therefore, these data cannot support a causal conclusion that CAR-T therapy is superior or inferior to BsAbs.

CAR-T therapy and BsAbs have different clinical advantages. CAR-T therapy is usually delivered as a one-time cellular therapy. If a deep response is achieved, it may provide a prolonged treatment-free interval. This is especially evident for cilta-cel, which has shown deep and durable responses in clinical trials (8, 9). In contrast, BsAbs usually require continuous or long-term administration. However, they are off-the-shelf agents. They do not require individualized cell collection or ex vivo expansion. They can be started more rapidly and may be useful for patients with rapidly progressive disease, those who cannot wait for CAR-T manufacturing, those who are not suitable for cellular therapy procedures, those without access to CAR-T therapy, or those who relapse after CAR-T therapy (10, 11). Thus, BsAbs should not be viewed simply as replacements for CAR-T therapy. They provide another T-cell–redirecting treatment option with different accessibility, toxicity profiles, and treatment schedules.

For safety, both CAR-T therapy and BsAbs are T-cell–redirecting therapies. Therefore, both can cause immune-related toxicities, including CRS, ICANS, cytopenias, and infections. CAR-T-related CRS and neurotoxicity usually occur early after cell infusion. BsAb-related CRS usually occurs during step-up dosing and the initial treatment cycles. In the MajesTEC-1 study, Moreau et al. reported that teclistamab-associated CRS was mostly low grade, although infections and cytopenias were common. Our results also showed that ICANS had a relatively low pooled incidence, whereas infection, grade ≥3 infection, and grade ≥3 neutropenia were common (14, 16, 17). These findings suggest that safety management during BsAb therapy should not focus only on acute CRS and ICANS. Infection prevention, recognition of hypogammaglobulinemia, routine blood count monitoring, IVIG supplementation, and management of myelosuppression are also critical during prolonged BsAb treatment, as emphasized in recent consensus recommendations and infection-focused studies (17–19, 36, 37).

Different BsAb targets may be associated with different toxicity profiles. It should be noted that the evidence base was imbalanced between BCMA×CD3 and GPRC5D×CD3 bispecific antibodies in this meta-analysis. The BCMA×CD3 group included several agents, such as teclistamab, elranatamab, alnuctamab, linvoseltamab, and ABBV-383. In contrast, the GPRC5D×CD3 group was mainly represented by talquetamab studies. Therefore, although target-based subgroup analyses suggested potential differences between BCMA×CD3 and GPRC5D×CD3 therapies for some efficacy and safety outcomes, these findings should be regarded as exploratory. They should not be interpreted as class-level effects for all GPRC5D-directed bispecific antibodies. More studies of different GPRC5D-directed agents, broader patient populations, and long-term real-world cohorts are needed to validate these findings.

BCMA is expressed not only on myeloma cells but also on normal plasma cells and is closely related to humoral immunity. BCMA-directed therapy may therefore increase infection risk through plasma cell depletion, hypogammaglobulinemia, and delayed immune reconstitution (5, 17–19). GPRC5D-directed therapy has a different target distribution. In addition to infection and hematologic toxicity, common on-target/off-tumor adverse events include dysgeusia, skin changes, and nail-related toxicities (6, 16). In our subgroup analysis, the incidence of grade ≥3 neutropenia was higher in the BCMA×CD3 subgroup than in the GPRC5D×CD3 subgroup. This suggests that drug target may be an important source of heterogeneity for hematologic toxicity. These results support individualized treatment selection based on prior therapy, infection risk, bone marrow reserve, target exposure, and tolerance for long-term continuous treatment.

Several outcomes showed substantial heterogeneity, especially ≥VGPR, CRS, infection, neutropenia, and grade ≥3 neutropenia. Sensitivity analyses showed that sequential omission of individual studies did not materially change the direction of the pooled effects. This suggests that the main findings were relatively stable. Meta-regression and subgroup analyses further suggested that drug target, drug family, study design, trial phase, and prior treatment exposure may be important sources of heterogeneity. For efficacy outcomes, ORR differed between BCMA×CD3 and GPRC5D×CD3 bispecific antibodies, suggesting that target selection may influence overall response. The ≥VGPR rate differed significantly between prospective clinical trials and retrospective real-world studies. This suggests that strict eligibility criteria, response assessment procedures, and completeness of follow-up may influence estimates of deep response. For safety outcomes, CRS and infection differed by study design and trial phase, whereas grade ≥3 neutropenia was associated with drug target and prior treatment background. These findings indicate that the efficacy and toxicity of BsAbs cannot be fully summarized by a single pooled estimate. They should be interpreted in the context of the specific agent, target, prior CAR-T or BCMA exposure, real-world patient characteristics, and supportive care strategies.

From a treatment-sequencing perspective, the optimal order of CAR-T therapy and BsAbs remains unclear. For eligible patients whose disease course allows time for manufacturing and who have access to a cellular therapy center, BCMA-directed CAR-T therapy, especially cilta-cel, may provide deep and durable responses (8, 9). For patients with rapidly progressive disease, those unable to wait for CAR-T manufacturing, those unsuitable for CAR-T procedures because of comorbidities or resource limitations, and those who relapse after CAR-T therapy, BsAbs provide an important treatment option. Prior exposure to BCMA-directed therapy may affect the efficacy of subsequent BCMA-directed BsAbs or CAR-T therapy. Therefore, non-BCMA strategies, such as GPRC5D×CD3 bispecific antibodies, may be particularly important after relapse following BCMA-directed therapy (6, 33, 38). The MajesTEC-1 cohort C study by Touzeau et al. showed that patients with prior BCMA-directed therapy could still benefit from teclistamab, although response depth and PFS may be lower than in BCMA-naïve patients (38). These findings highlight the need for further studies to define the optimal sequence of BCMA CAR-T therapy, BCMA×CD3 BsAbs, and GPRC5D×CD3 BsAbs (39, 40), as well as the mechanisms of cross-resistance.

## 5 Limitations

This study has several limitations. First, the included studies were mainly single-arm phase I/II clinical trials and retrospective real-world cohorts. No randomized controlled studies were available. Therefore, we could not directly compare efficacy or safety across different BsAbs, or between BsAbs and CAR-T-cell therapy. Second, although we discussed our findings in the context of CAR-T studies such as ide-cel and cilta-cel, this comparison was indirect and contextual. It was affected by differences in eligibility criteria, number of prior lines of therapy, BCMA exposure status, follow-up duration, and endpoint assessment. Therefore, these data cannot support causal conclusions about whether CAR-T therapy is superior or inferior to BsAbs.

Third, the included studies differed in drug target, drug family, dosing regimen, step-up dosing strategy, CRS management, infection prophylaxis, intravenous immunoglobulin (IVIG) use, and follow-up duration. These differences contributed to substantial heterogeneity in several outcomes. Fourth, real-world studies and clinical trials differed in performance status, comorbidities, tumor burden, prior treatment exposure, and completeness of response assessment. These differences may have affected the pooled estimates. Fifth, some outcomes were based on a limited number of cohorts, especially sCR, PFS, and grade ≥3 thrombocytopenia. This limited the precision of these estimates.

Sixth, this review was not prospectively registered before study screening and data extraction. This may reduce methodological transparency and increase the risk of selective reporting. In addition, ClinicalTrials.gov and other trial registries were not systematically searched as independent sources, which may have led to omission of unpublished or ongoing studies. To mitigate these limitations, we provided the full search strategy, detailed data extraction tables, sensitivity analyses, meta-regression, subgroup analyses, publication-bias assessments, and GRADE certainty-of-evidence assessment. Seventh, although the MINORS assessment showed that the overall methodological quality of the included studies was moderate to high, most studies were still single-arm, open-label clinical trials or retrospective real-world cohorts. They lacked randomized control groups and may still be affected by selection bias, information bias, and residual confounding. The GRADE assessment also showed that the certainty of evidence for most major outcomes was low to very low. Therefore, our findings should be interpreted with caution. Future head-to-head comparative studies, randomized controlled trials, and long-term real-world studies are needed.

In addition, recurrent infections, infection prophylaxis, IVIG supplementation, corticosteroid exposure, hospitalization, treatment interruption, and treatment discontinuation were not consistently reported across studies. Therefore, we could not perform reliable pooled analyses for these more granular infection-related outcomes. The pooled infection estimates should be considered descriptive rather than uniform risk estimates applicable to all clinical settings.

## 6 Conclusion

In summary, BCMA×CD3 and GPRC5D×CD3 bispecific antibodies showed high overall response rates and meaningful deep responses in patients with RRMM. They provide an important treatment option for patients who are not suitable for CAR-T-cell therapy, cannot receive CAR-T-cell therapy in a timely manner, or relapse after CAR-T-cell or BCMA-directed therapy. Compared with CAR-T-cell therapies such as ide-cel and cilta-cel, BsAbs may show less durable and less deep responses than those reported in some CAR-T studies, especially cilta-cel. However, their off-the-shelf availability, convenient administration, and potential role in treatment sequencing make them clinically valuable in the RRMM treatment landscape. Future studies should define the optimal sequencing of CAR-T therapy and BsAbs, clarify cross-resistance mechanisms after different target-directed therapies, improve infection and cytopenia risk management, and develop individualized treatment strategies for different patient populations.

## Data Availability

The original contributions presented in the study are included in the article/**Supplementary Material**. Further inquiries can be directed to the corresponding authors.
