# Correction: Efficacy and safety of BCMA-or GPRC5D-directed CD3 bispecific antibodies in relapsed/refractory multiple myeloma: a systematic review and meta-analysis of prospective clinical trials and real-world studies

**DOI:** 10.3389/fimmu.2026.1911100

**Published:** 2026-06-22

**Authors:** Jiashun Li, Ainikaer Abulaiti, Deyu Li, Yan Zhao, Paerhati Wahafu, Maerdan Maimaitiming, Shi Qiu, Yuxiang Zhang, Yaqin An, Wenxing Wang, Liangquan Shi, Maihemuti Yakufu, Li Shu, Guohua Li, Zhen Liu

**Affiliations:** 1 Department of Sports Medicine, Sixth Affiliated Hospital of Xinjiang Medical University, Urumqi, China; 2 Department Laboratory, The Six Affiliated Hospital, Xinjiang Medical University, Urumqi, Xinjiang, China

**Keywords:** BCMA, bispecific antibodies, GPRC5D, meta-analysis, multiple myeloma

In the published article, there was an error in the **Funding** statement. The **Funding** statement was incorrectly published as:

“This work was financially supported by the Health Care and Medical Research Special Project of Xinjiang Uygur Autonomous Region (BL202675, BL202573, BL202460), Youth Medical Science and Technology Talent Special Research Project (WJWY-202331), National Natural Science Foundation of China (82460420), Research and Innovation Team Project, Xinjiang Medical University (XYD2024C12), Tianshan Talent Training Program (TSYC202301B039, TSYC202301B077 and TSYC202301B106), Key Laboratory of High Incidence Disease Research in Xinjiang (Xinjiang Medical University), Ministry of Education (2023B04), Natural Science.”

The correct **Funding** statement reads:

“This work was financially supported by the Health Care and Medical Research Special Project of Xinjiang Uygur Autonomous Region (BL202675, BL202573, BL202460), Youth Medical Science and Technology Talent Special Research Project (WJWY-202331), National Natural Science Foundation of China (82460420), Research and Innovation Team Project, Xinjiang Medical University (XYD2024C12), Tianshan Talent Training Program (TSYC202301B039, TSYC202301B077 and TSYC202301B106), Key Laboratory of High Incidence Disease Research in Xinjiang (Xinjiang Medical University), Ministry of Education (2023B04), Natural Science Foundation of Xinjiang Uygur Autonomous Region (2022D01C821), Research and Innovation Team Project, Sixth Affiliated Hospital of Xinjiang Medical University (LFYKYZXJJ2024001), Tianshan Innovation Team Program (2025D14019), and Tianshan District Science & Technology Development Program (WLMQTSQ-2025-KJXM05).”

The original version of this article has been updated.

This correction does not change the scientific conclusions of the article in any way.

