# Supplementary material for: Efficacy and safety of BCMA- or GPRC5D-directed CD3 bispecific antibodies in relapsed/refractory multiple myeloma: a systematic review and meta-analysis of prospective clinical trials and real-world studies
Source: Front Immunol. 2026 May 20;17:1811816. doi: 10.3389/fimmu.2026.1811816 (PMC13230190; doi:10.3389/fimmu.2026.1811816)
Supplement: Supplementary file 1 [file DataSheet1.zip › Supplementary File2 Study Characteristics.docx]

| Study | Trial number | Phase of trial | Assessable size | Previous treatment | Intervention | Drug target | Male(%) | Median age, years | HRCA(%) | Median follow-up duration, months |
| --- | --- | --- | --- | --- | --- | --- | --- | --- | --- | --- |
| Al Hadidi.2025**^[19]^** | N/A | Retrospective | 114 | All patients were triple-class refractory, had a median of 6 prior therapies, and 65% had received prior BCMA-targeted therapy. | Patients received talquetamab at a dose of 0.8 mg/kg subcutaneously with a step‑up dosing schedule. | GPRC5D×CD3 | 56 | 67 (34–90) | 70 | 6.7 (0.2-15.9) |
| An.2025**A^[20]^** | NCT03399799 and NCT04634552 | II (MonumenTAL-1 China cohort) | 29 | Patients had relapsed/refractory multiple myeloma, had received 3 or more prior lines of therapy including ≥1 proteasome inhibitor, ≥1 immunomodulatory drug, and ≥1 anti-CD38 monoclonal antibody, and had no prior exposure to T-cell redirecting therapy. Baseline disease characteristics in the QW cohort included extramedullary disease in 20.7%, high-risk cytogenetics in 37.0%, and ISS stage III disease in 6.9%. | Patients received subcutaneous talquetamab 0.4 mg/kg weekly with protocol-specified pretreatment using dexamethasone, an antihistamine, and an antipyretic; the article states the regimen was given with two to three step-up doses. | GPRC5D×CD3 | NR | NR | 37 | 16.3 |
| An.2025**B^[20]^** | NCT03399799 and NCT04634552 | II (MonumenTAL-1 China cohort) | 12 | Patients had relapsed/refractory multiple myeloma, had received 3 or more prior lines of therapy including ≥1 proteasome inhibitor, ≥1 immunomodulatory drug, and ≥1 anti-CD38 monoclonal antibody, and had no prior exposure to T-cell redirecting therapy. Baseline disease characteristics in the Q2W cohort included extramedullary disease in 8.3%, high-risk cytogenetics in 30.0%, and ISS stage III disease in 8.3%. | Patients received subcutaneous talquetamab 0.8 mg/kg every 2 weeks with protocol-specified pretreatment using dexamethasone, an antihistamine, and an antipyretic; the article states the regimen was given with two to three step-up doses. | GPRC5D×CD3 | NR | NR | 30 | 8.2 |
| Bahlis.2023**^[21]^** | NCT03269136 | Ⅰ | 55 | All patients were triple-class exposed, 90.9% were triple-class refractory, and 23.6% had received prior BCMA-targeted therapy. | Patients received elranatamab at doses ≥215 µg/kg up to 1000 µg/kg subcutaneously, either with a single priming dose or with priming plus premedication. | BCMA×CD3 | 52.7 | 64 (42–80) | 29.1 | 12 (0.3-32.3) |
| Bar.2026**^[22]^** | NCT03486067 | I | 49 | All patients were triple-class exposed, had a median of 4 prior therapies, were BCMA-therapy naïve, and 64.2% were triple-class refractory. | Patients received alnuctamab at target doses of 10–60 mg subcutaneously following 3 mg and 6 mg step‑up doses, with subsequent de‑escalation to less frequent dosing. | BCMA×CD3 | 57.1 | 64 (36-85) | 30.6 | 11.8 |
| Bumma.2024**^[23]^** | NCT03761108 | I/II | 117 | All patients received triple-class therapy (PI/IMiD/anti-CD38), 77% were penta-exposed, and the study excluded those with prior BCMA-targeted immunotherapy. | Patients received linvoseltamab at doses of 50 mg or 200 mg intravenously with a step‑up dosing regimen. | BCMA×CD3 | 54.7 | 70 (37-91) | 39.3 | 14.3 |
| Chari.2025**A^[15]^** | NCT03399799 and NCT04634552 | MonumenTAL-1, phase 1-2 | 143 | All patients were TCR-naive and had relapsed/refractory multiple myeloma after at least 3 previous lines of therapy including a proteasome inhibitor, an immunomodulatory drug, and an anti-CD38 monoclonal antibody; 100% were triple-class exposed, 73% were penta-drug exposed, 75% were triple-class refractory, 31% were penta-drug refractory, and 94% were refractory to the last line of therapy. | Patients received subcutaneous talquetamab 0.4 mg/kg once weekly with step-up doses of 0.01 mg/kg and 0.06 mg/kg. | GPRC5D×CD3 | 55 | 67 (58–72) | 31 | 25.6 (8.5–25.9) |
| Chari.2025**B^[15]^** | NCT03399799 and NCT04634552 | MonumenTAL-1, phase 1-2 | 154 | All patients were TCR-naive and had relapsed/refractory multiple myeloma after at least 3 previous lines of therapy including a proteasome inhibitor, an immunomodulatory drug, and an anti-CD38 monoclonal antibody; 100% were triple-class exposed, 69% were penta-drug exposed, 71% were triple-class refractory, 25% were penta-drug refractory, and 94% were refractory to the last line of therapy. | Patients received subcutaneous talquetamab 0.8 mg/kg every 2 weeks with step-up doses of 0.01 mg/kg, 0.06 mg/kg, and 0.3 mg/kg. | GPRC5D×CD3 | 58 | 67 (58–74) | 30 | 19.4 (9.2–20.7) |
| D'Souza.2022**^[24]^** | NCT03933735 | I | 79 | All patients received triple-class therapy (PI/IMiD/anti-CD38), 91% were penta-exposed, 35% were penta-drug refractory, and the study excluded those with prior BCMA-targeted therapy. | Patients received ABBV-383 at doses ranging from 0.025 to 120 mg via intravenous infusion every 3 weeks. | BCMA×CD3 | 55 | 68 (35-92) | 14 | 10.8 (0.6-28.2) |
| Frenking.2025**^[25]^** | N/A | Retrospective | 123 | All patients received triple-class therapy (PI/IMiD/anti-CD38), 77% were penta-exposed, 47% were penta-drug refractory, 20% had prior BTCE exposure, and 34% had prior CAR-T therapy. | Patients received talquetamab, primarily at a dose of 800 μg/kg administered subcutaneously every 2 weeks. | GPRC5D×CD3 | 70 | 64 (24-84) | 48 | 7.8 |
| Lesokhin.2023**^[14]^** | NCT04649359 | II | 123 | All patients received triple-class therapy (PI/IMiD/anti-CD38), 96.7% were triple-class refractory, 42.3% were penta-drug refractory, and the study excluded those with prior BCMA-directed therapy. | Patients received subcutaneous elranatamab 76 mg weekly after two step-up priming doses (12 mg and 32 mg) in cycle 1, with responders switching to biweekly dosing after six cycles. | BCMA×CD3 | 55.3 | 68 (36-89) | 25.2 | 14.7 (0.2-25.1) |
| Mohan.2024**^[26]^** | N/A | Retrospective | 110 | All patients received triple-class therapy (PI/IMiD/anti-CD38), 76% were penta-drug refractory, 35% had prior BCMA-targeted therapy. | Patients received standard-of-care teclistamab with step-up doses of 0.06 mg/kg, 0.3 mg/kg, and 1.5 mg/kg, followed by maintenance therapy at 1.5 mg/kg weekly. | BCMA×CD3 | 50.9 | 68 (37-89) | 62 | 3.5 (0.39-10.92) |
| Moreau.2022**^[13]^** | NCT03145181 and NCT04557098 | MajesTEC-1 phase 1/2 | 165 | All patients had relapsed/refractory multiple myeloma after at least 3 prior lines of therapy and were triple-class exposed (IMiD/PI/anti-CD38); median prior lines of therapy was 5, 70.3% were penta-drug exposed, 77.6% had triple-class refractory disease, 30.3% had penta-drug refractory disease, and prior BCMA-targeted therapy was not allowed. | Patients received once-weekly subcutaneous teclistamab at 1.5 mg/kg after step-up doses of 0.06 mg/kg and 0.3 mg/kg. | BCMA×CD3 | 58.2 | 64 (33–84) | 25.7 | 14.1 (0.3–24.4) |
| Razzo.2025**^[27]^** | N/A | Retrospective | 509 | Patients had relapsed/refractory multiple myeloma and were heavily pretreated; median prior lines of therapy was 6 (2–18), 81% were triple-class refractory, 38% were penta-drug refractory, and 236 patients had prior BCMA-directed therapy. | Patients received teclistamab according to each center’s local standard of care, including step-up dosing protocols and response assessments; eligible patients had received at least one full dose of teclistamab through commercial access or an expanded access program. | BCMA×CD3 | 54 | 68 (31–92) | 54 | 10.1 |
| Shigeki.2025**^[28]^** | NCT03399799 and NCT04634552 | II (MonumenTAL-1 Japan cohort) | 36 | Patients had relapsed/refractory multiple myeloma and no prior exposure to T-cell redirection therapies; all were triple-class exposed, median prior lines of therapy was 3.5 (3–13), 58.3% were penta-drug exposed, 69.4% were triple-class refractory, 22.2% were penta-drug refractory, and 97.2% were refractory to the last line of therapy. | Patients received subcutaneous talquetamab 0.4 mg/kg once weekly with two step-up doses of 0.01 mg/kg and 0.06 mg/kg, plus pretreatment medications before step-up doses and the initial full treatment dose. | GPRC5D×CD3 | 55.6 | 70.5 (46–81) | 39.4 | 13.4 (0.6–24.7) |
| Touzeau.2024**^[29]^** | NCT03145181 and NCT04557098 | I/II (MajesTEC-1 cohort C) | 40 | All patients had relapsed/refractory multiple myeloma, were triple-class exposed, and had prior exposure to anti-BCMA therapy; median prior lines of therapy was 6 (range, 3–14), 80.0% were penta-drug exposed, 85.0% were triple-class refractory, 35.0% were penta-drug refractory, and 67.5% were refractory to prior BCMA-directed therapy. Prior anti-BCMA therapy included ADC (n=29), CAR-T (n=15), or both (n=4). | Patients received subcutaneous teclistamab at 1.5 mg/kg weekly after step-up doses of 0.06 mg/kg and 0.3 mg/kg, with the option to switch to every-2-weeks dosing after sustained CR or better. | BCMA×CD3 | 62.5 | 63.5 (32–82) | 33.3 | 28.0 (0.7–31.1) |
| Yi.2025**^[30]^** | N/A | Retrospective | 42 | Patients had a median of 6 prior lines of therapy, were all exposed to proteasome inhibitors, immunomodulatory drugs, and anti-CD38 monoclonal antibodies, with a subset being triple- or penta-class refractory. | Patients received teclistamab with step-up doses of 0.06 mg/kg and 0.3 mg/kg, followed by maintenance therapy at 1.5 mg/kg weekly. | BCMA×CD3 | 64.3 | 60 (43-80) | 23.8 | 16.4 (15..1-17.7) |
