# Supplementary material for: Efficacy and safety of BCMA- or GPRC5D-directed CD3 bispecific antibodies in relapsed/refractory multiple myeloma: a systematic review and meta-analysis of prospective clinical trials and real-world studies
Source: Front Immunol. 2026 May 20;17:1811816. doi: 10.3389/fimmu.2026.1811816 (PMC13230190; doi:10.3389/fimmu.2026.1811816)
Supplement: Supplementary file 1 [file DataSheet1.zip › Supplementary File3 Quality Assessment.docx]

***Supplementary File 3. GRADE Evidence Profile and Summary of Findings***

*Bispecific antibodies targeting BCMA×CD3 or GPRC5D×CD3 in relapsed/refractory multiple myeloma*

This table was prepared according to the GRADE framework. The initial certainty of evidence was rated as low because the included evidence mainly consisted of single-arm prospective clinical trials and retrospective real-world cohorts without randomized comparator groups. Publication bias was rated as not serious for all outcomes based on the publication-bias assessments provided in Supplementary File 8.

| **Outcome** | **Cohorts (n)** | **Pooled estimate** | **I²** | **Risk of bias** | **Inconsistency** | **Indirectness** | **Imprecision** | **Publication bias** | **Certainty of evidence** |
| --- | --- | --- | --- | --- | --- | --- | --- | --- | --- |
| PFS | 6 | 8.76 months 95% CI 6.82-11.13 | 59.96% | Serious | Serious | Serious | Serious | Not serious | Very low |
| sCR | 4 | 28.7% 95% CI 19.2-40.6 | 66.64% | Serious | Serious | Not serious | Serious | Not serious | Very low |
| CR | 6 | 18.1% 95% CI 14.7-22.3 | 44.28% | Not serious | Not serious | Not serious | Not serious | Not serious | Low |
| ≥CR | 12 | 38.2% 95% CI 33.0-43.5 | 63.84% | Not serious | Serious | Serious | Not serious | Not serious | Very low |
| ≥VGPR | 15 | 51.0% 95% CI 44.0-57.7 | 83.67% | Not serious | Very serious | Serious | Not serious | Not serious | Very low |
| ORR | 17 | 65.9% 95% CI 62.2-69.4 | 56.85% | Not serious | Not serious | Not serious | Not serious | Not serious | Low |
| CRS | 17 | 67.5% 95% CI 61.1-73.5 | 85.99% | Not serious | Very serious | Serious | Not serious | Not serious | Very low |
| ICANS | 14 | 9.7% 95% CI 8.3-11.3 | 39.01% | Not serious | Not serious | Not serious | Not serious | Not serious | Low |
| Neutropenia | 14 | 54.7% 95% CI 45.5-63.4 | 87.89% | Not serious | Very serious | Serious | Not serious | Not serious | Very low |
| Grade ≥3 neutropenia | 14 | 42.6% 95% CI 33.2-52.5 | 89.77% | Not serious | Very serious | Serious | Not serious | Not serious | Very low |
| Infection | 16 | 60.3% 95% CI 52.0-67.9 | 90.25% | Not serious | Very serious | Serious | Not serious | Not serious | Very low |
| Grade ≥3 infection | 15 | 38.3% 95% CI 31.6-44.9 | 63.84% | Not serious | Serious | Serious | Not serious | Not serious | Very low |
| Grade ≥3 anemia | 13 | 30.4% 95% CI 25.5-35.7 | 65.10% | Not serious | Serious | Serious | Not serious | Not serious | Very low |
| Grade ≥3 thrombocytopenia | 7 | 21.9% 95% CI 16.3-28.3 | 73.33% | Serious | Serious | Serious | Serious | Not serious | Very low |

**Abbreviations:** GRADE, Grading of Recommendations Assessment, Development and Evaluation; CI, confidence interval; PFS, progression-free survival; sCR, stringent complete response; CR, complete response; VGPR, very good partial response; ORR, overall response rate; CRS, cytokine release syndrome; ICANS, immune effector cell-associated neurotoxicity syndrome.

**Footnotes:**

a. The initial certainty of evidence was rated as low because the included evidence mainly consisted of single-arm prospective clinical trials and retrospective real-world cohorts without randomized comparator groups.

b. Risk of bias was not further downgraded for most outcomes because the included studies generally had clearly defined objectives, prespecified clinical endpoints, and extractable numerator/denominator data. PFS, sCR, and grade ≥3 thrombocytopenia were downgraded for study limitations because of limited contributing cohorts, differences in follow-up duration, and potential variability in outcome ascertainment.

c. Inconsistency was downgraded when substantial heterogeneity was observed. Outcomes with I² >50% were generally downgraded for serious inconsistency, while outcomes with I² >75% were downgraded for very serious inconsistency.

d. Indirectness was downgraded when pooled estimates were influenced by differences in drug target, drug family, study design, trial phase, real-world versus clinical trial setting, or prior BCMA-directed/T-cell redirecting therapy exposure.

e. Imprecision was downgraded when the number of contributing cohorts was small, confidence intervals were relatively wide, or the outcome was vulnerable to sparse-event bias.

f. Publication bias was considered not serious for all outcomes because Supplementary File 8 provided publication-bias assessments for all prespecified efficacy and safety outcomes. For outcomes with fewer contributing cohorts, publication-bias assessments were mainly qualitative and should be interpreted cautiously.

g. Sensitivity analyses, subgroup analyses, and meta-regression were considered when judging inconsistency and indirectness. Although these analyses partly explained heterogeneity for several outcomes, residual heterogeneity remained for some safety outcomes.

**Methodological quality assessment using the MINORS criteria**

The Methodological Index for Non-Randomized Studies (MINORS) was used to assess the methodological quality of the included non-comparative studies. Each item was scored as 0 (not reported), 1 (reported but inadequate), or 2 (reported adequately). The maximum score for non-comparative studies is 16.

| **Study** | **Study design** | **I1** | **I2** | **I3** | **I4** | **I5** | **I6** | **I7** | **I8** | **Total** | **Quality** |
| --- | --- | --- | --- | --- | --- | --- | --- | --- | --- | --- | --- |
| Al Hadidi.2025 | Retrospective real-world | 2 | 2 | 0 | 2 | 2 | 2 | 1 | 0 | 11 | Moderate |
| An.2025 | Prospective phase 1/2 cohort | 2 | 2 | 2 | 2 | 1 | 2 | 2 | 1 | 14 | High |
| Bahlis.2023 | Prospective phase 1 | 2 | 1 | 2 | 2 | 1 | 2 | 2 | 0 | 12 | Moderate |
| Bar.2026 | Prospective phase 1 | 2 | 1 | 2 | 2 | 1 | 2 | 2 | 0 | 12 | Moderate |
| Bumma.2024 | Prospective phase 1/2 | 2 | 2 | 2 | 2 | 1 | 2 | 2 | 1 | 14 | High |
| Chari.2025 | Prospective phase 1/2 | 2 | 2 | 2 | 2 | 1 | 2 | 2 | 1 | 14 | High |
| D'Souza.2022 | Prospective phase 1 | 2 | 1 | 2 | 2 | 1 | 2 | 2 | 0 | 12 | Moderate |
| Frenking.2025 | Retrospective real-world | 2 | 2 | 0 | 2 | 2 | 2 | 1 | 0 | 11 | Moderate |
| Lesokhin.2023 | Prospective phase 2 | 2 | 2 | 2 | 2 | 1 | 2 | 2 | 1 | 14 | High |
| Mohan.2024 | Retrospective real-world | 2 | 2 | 0 | 2 | 2 | 1 | 2 | 0 | 11 | Moderate |
| Moreau.2022 | Prospective phase 1/2 | 2 | 2 | 2 | 2 | 1 | 2 | 2 | 1 | 14 | High |
| Razzo.2025 | Retrospective real-world | 2 | 2 | 0 | 2 | 2 | 2 | 1 | 0 | 11 | Moderate |
| Shigeki.2025 | Prospective clinical cohort | 2 | 2 | 2 | 2 | 1 | 2 | 2 | 1 | 14 | High |
| Touzeau.2024 | Prospective phase 1/2 cohort | 2 | 2 | 2 | 2 | 1 | 2 | 2 | 1 | 14 | High |
| Yi.2025 | Retrospective real-world | 2 | 2 | 0 | 2 | 2 | 2 | 1 | 0 | 11 | Moderate |

**MINORS items:** I1, a clearly stated aim; I2, inclusion of consecutive patients; I3, prospective collection of data; I4, endpoints appropriate to the aim of the study; I5, unbiased assessment of the study endpoint; I6, follow-up period appropriate to the aim of the study; I7, loss to follow-up less than 5%; I8, prospective calculation of the study size.

Quality interpretation: high quality, 13–16 points; moderate quality, 9–12 points; low quality, 0–8 points. This threshold was used only to facilitate interpretation of the MINORS scores.

Abbreviations: MINORS, Methodological Index for Non-Randomized Studies; BCMA, B-cell maturation antigen; GPRC5D, G protein-coupled receptor class C group 5 member D; RRMM, relapsed/refractory multiple myeloma.

*Note: For studies with separately analyzable cohorts derived from the same trial or report, MINORS was assessed at the study/report level rather than the cohort level.*
