# Supplementary material for: Efficacy and safety of BCMA- or GPRC5D-directed CD3 bispecific antibodies in relapsed/refractory multiple myeloma: a systematic review and meta-analysis of prospective clinical trials and real-world studies
Source: Front Immunol. 2026 May 20;17:1811816. doi: 10.3389/fimmu.2026.1811816 (PMC13230190; doi:10.3389/fimmu.2026.1811816)
Supplement: Supplementary file 1 [file DataSheet1.zip › Supplementary File4 Sensitivity analysis.docx]

***Supplementary File4: Sensitivity analysis***


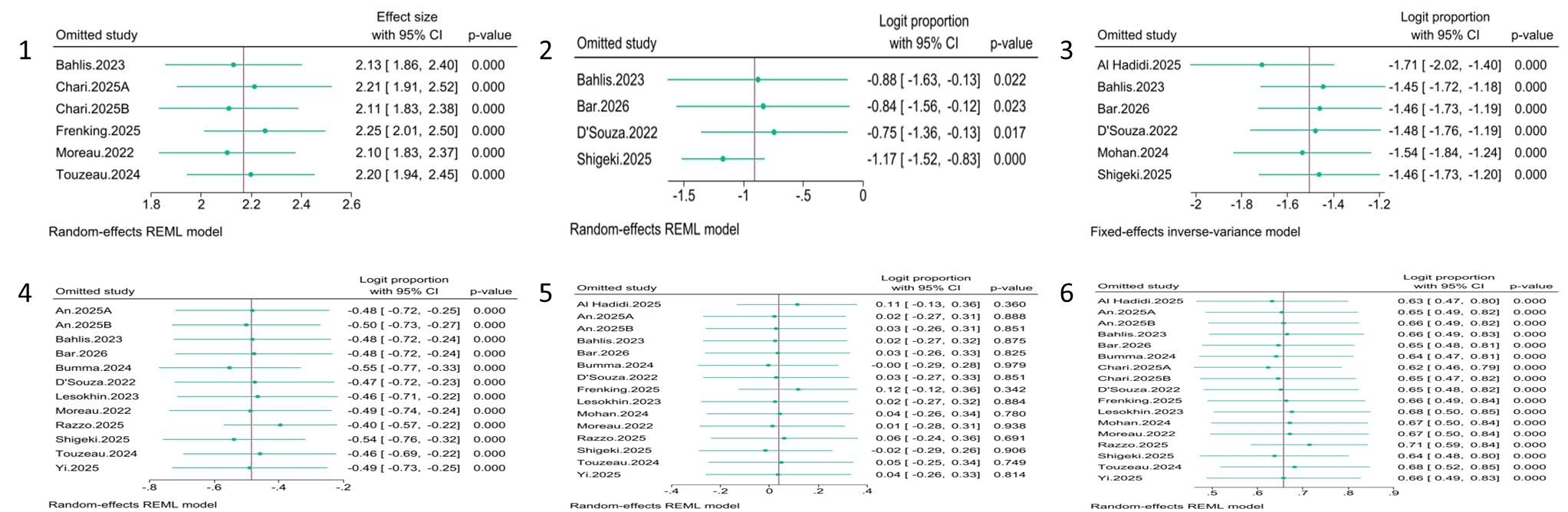


**Figure5. Sensitivity analysis of efficacy-related outcome indicators:** 1: PFS, 2: sCR, 3: CR, 4: ≥CR, 5: ≥VGPR, 6: ORR.


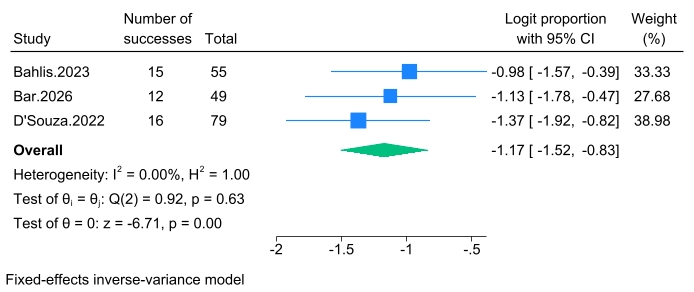


**Figure6:** sCR analysis after excluding the studies of Shigeki et al.


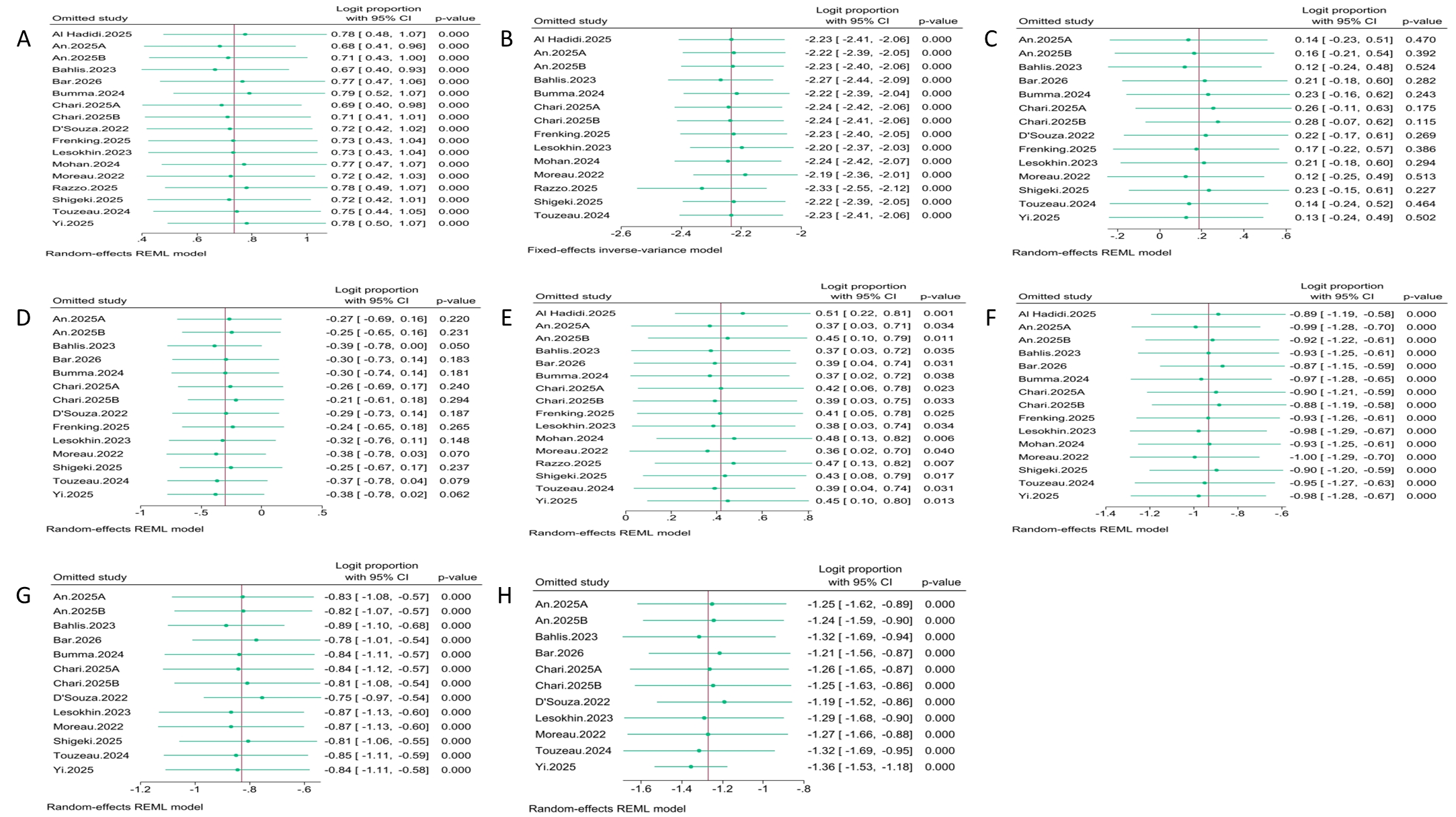


**Figure7. Sensitivity Analysis of Adverse Reaction-Related Outcome Indicators:** A: CRS, B: ICANS, C: Neutropenia, D: Grade ≥3 Neutropenia, E: Infection, F: Grade ≥3 infection, G: Grade ≥3 Anemia, H: Grade ≥3 Thrombocytopenia
