# Supplementary material for: Efficacy and safety of BCMA- or GPRC5D-directed CD3 bispecific antibodies in relapsed/refractory multiple myeloma: a systematic review and meta-analysis of prospective clinical trials and real-world studies
Source: Front Immunol. 2026 May 20;17:1811816. doi: 10.3389/fimmu.2026.1811816 (PMC13230190; doi:10.3389/fimmu.2026.1811816)
Supplement: Supplementary file 1 [file DataSheet1.zip › Supplementary File5 Meta regression.docx]

***Supplementary File 5: Meta regression***


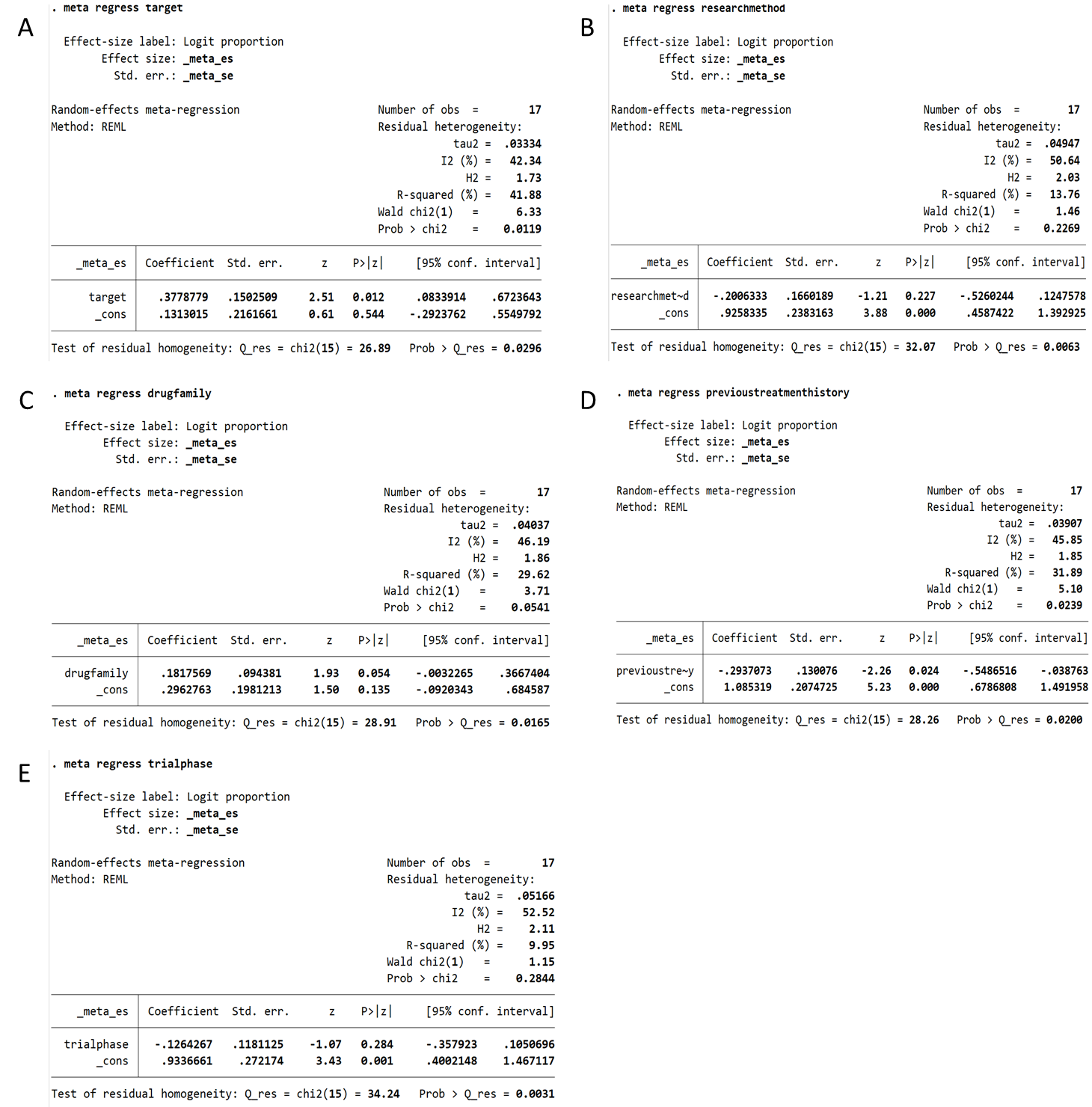


Figure8: Meta regression of ORR.


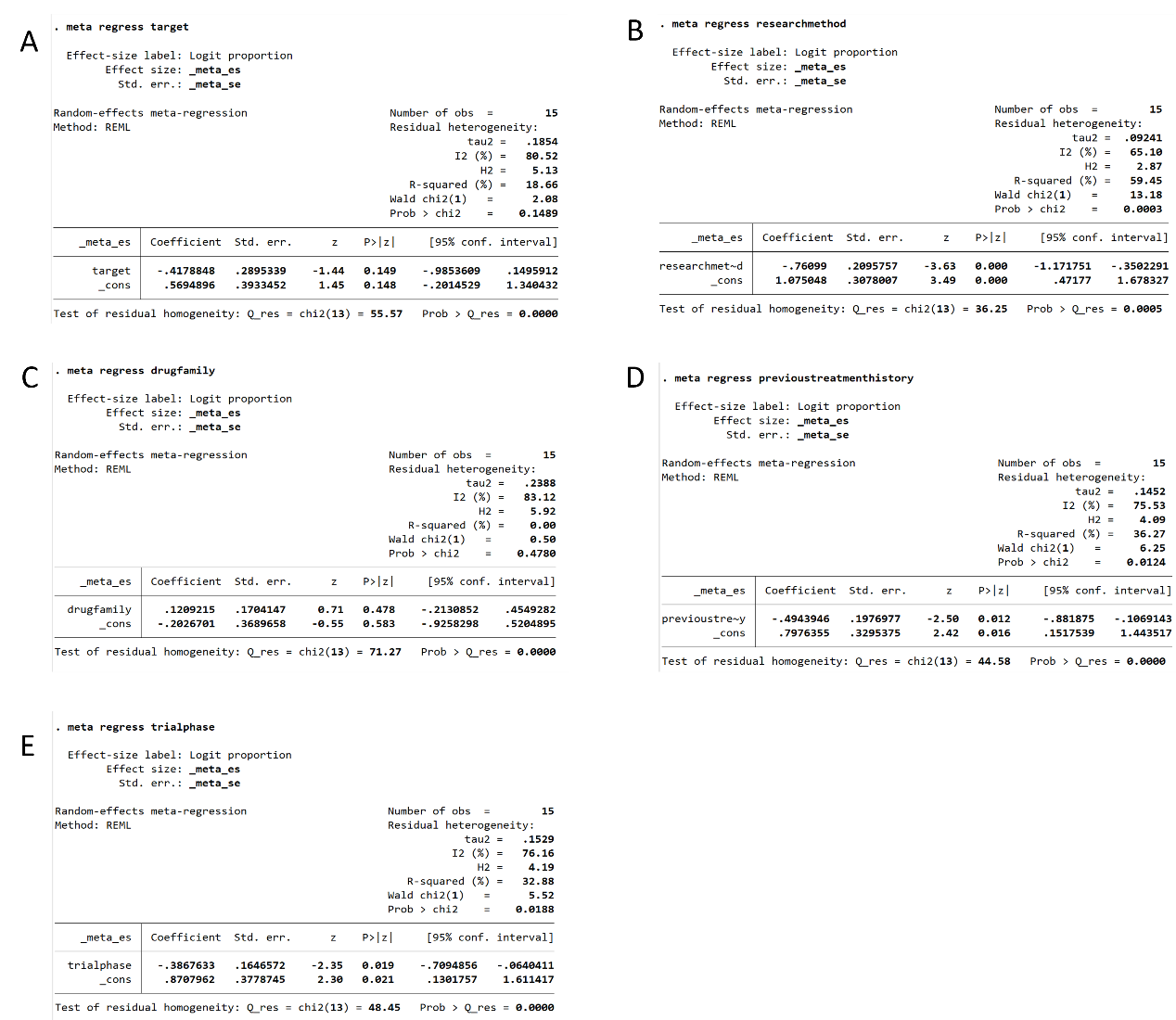


Figure9: Meta regression of ≥VGPR.


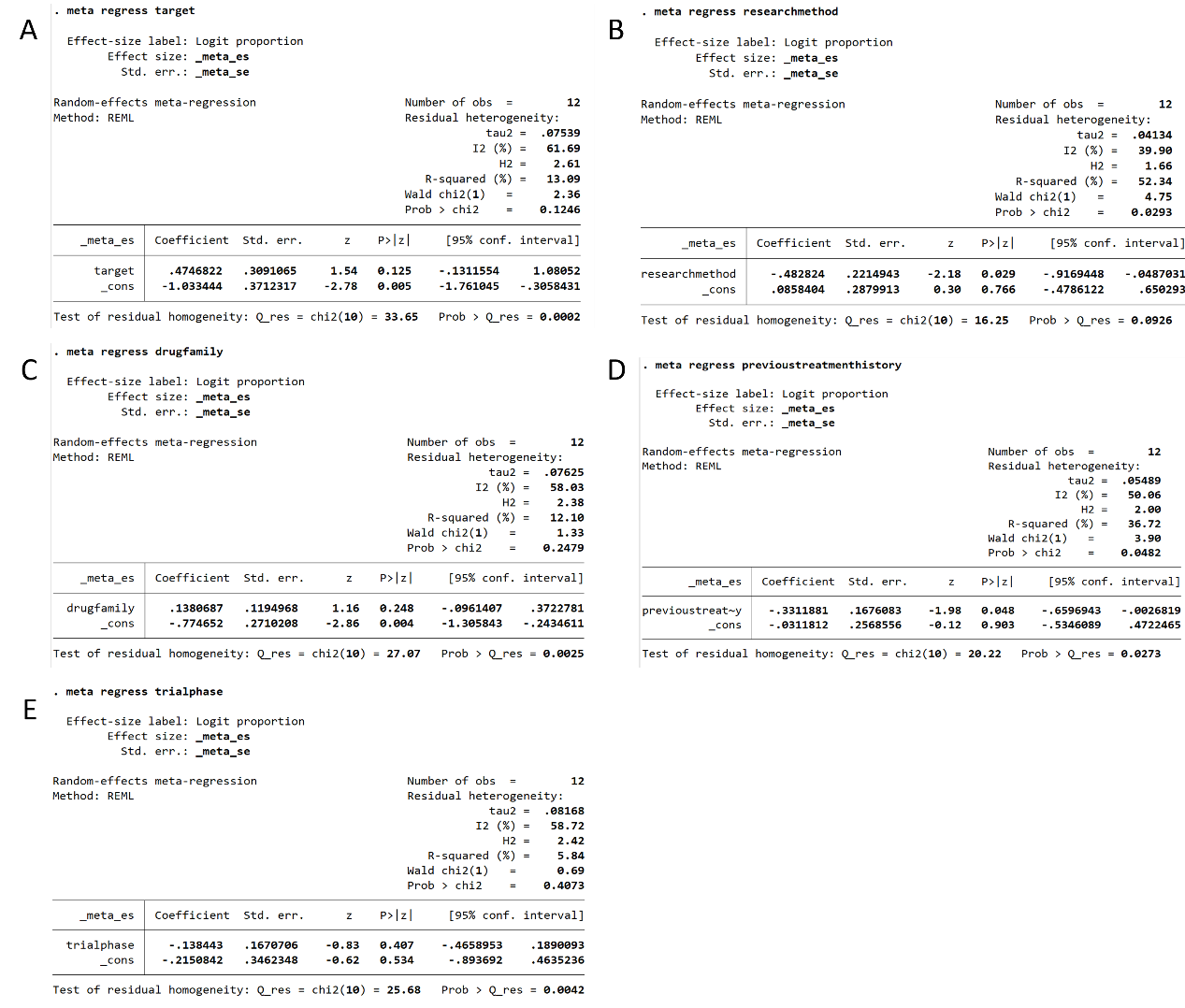


Figure10: Meta regression of ≥CR.


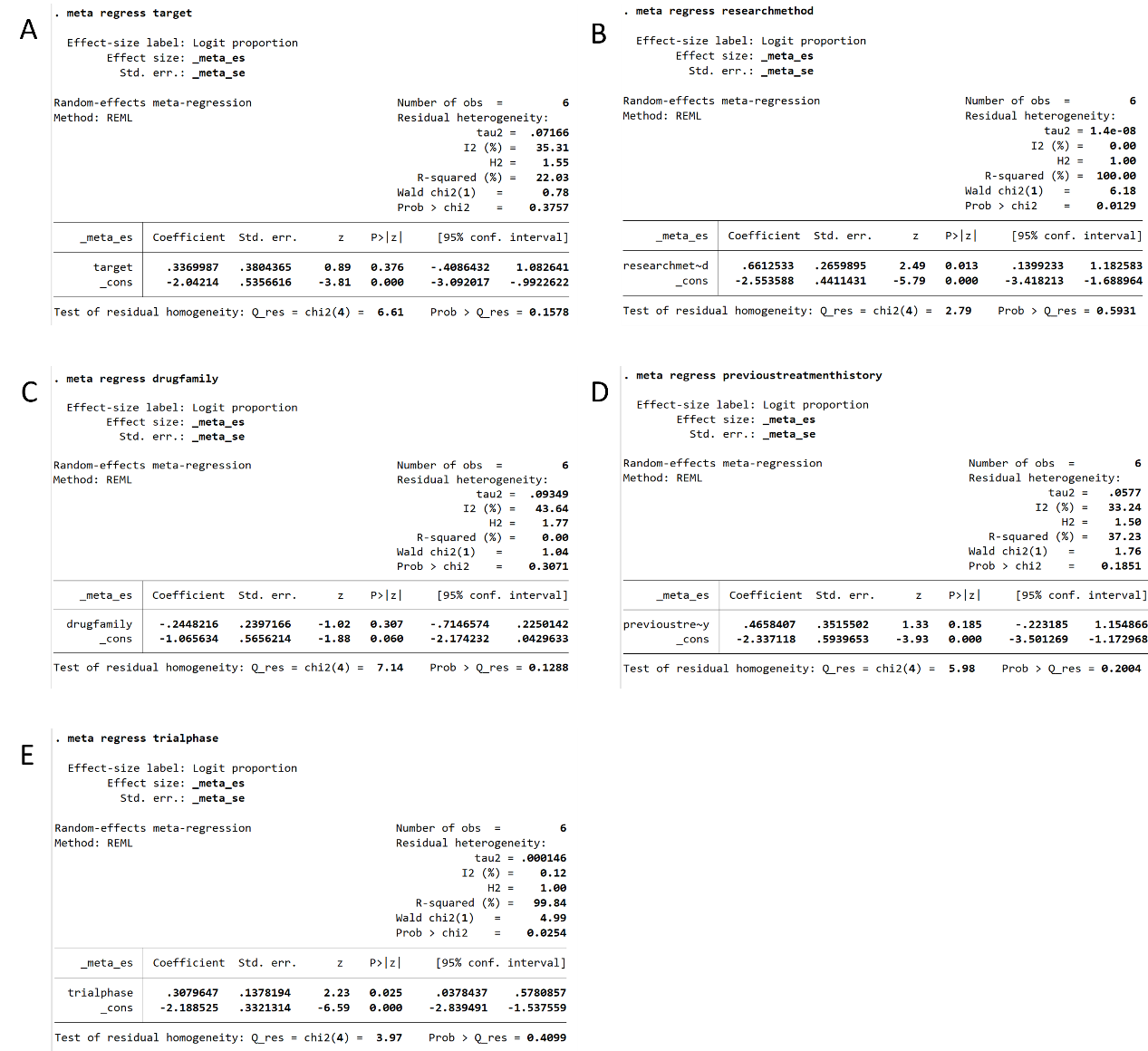


Figure11: Meta regression of CR.


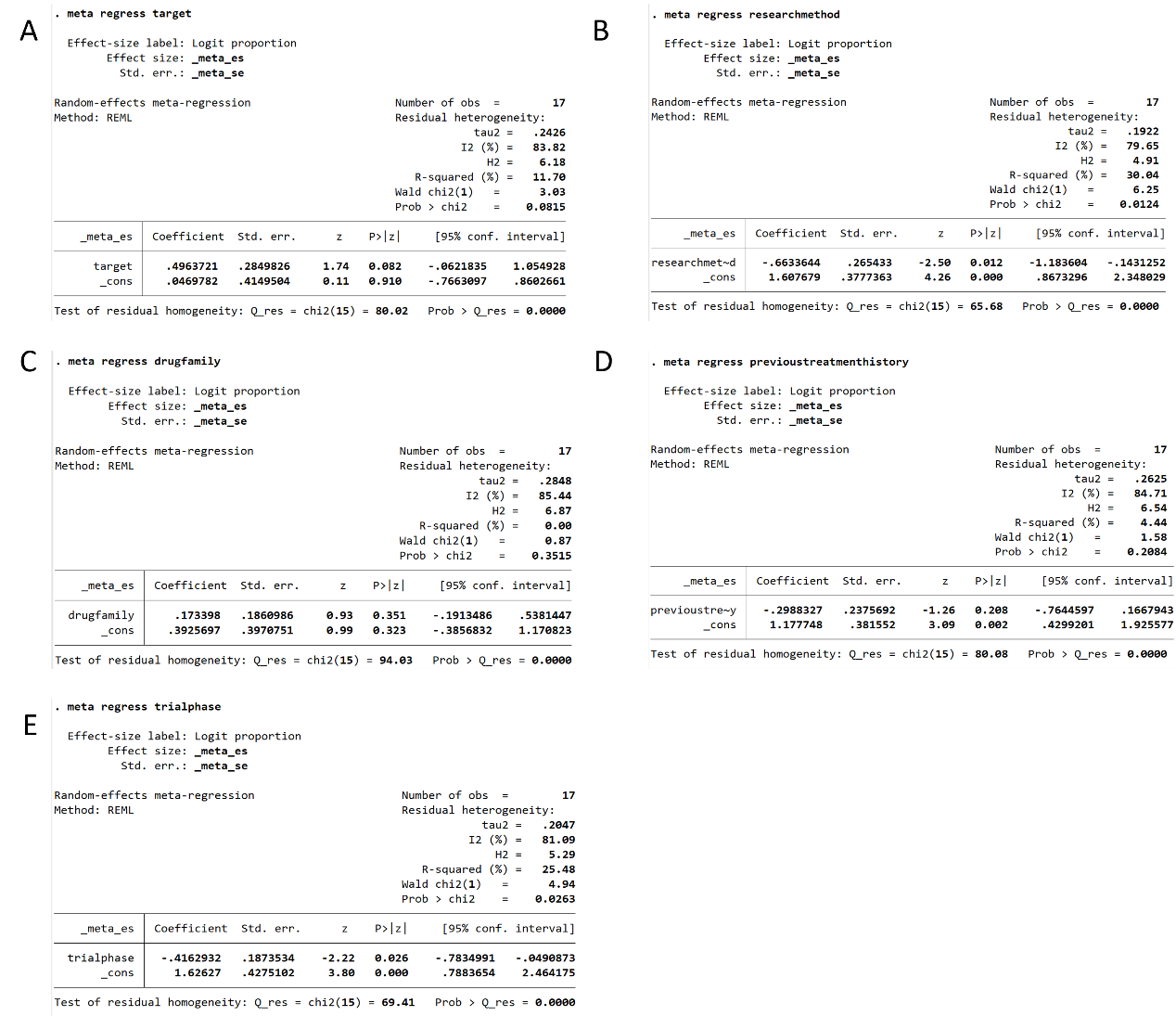


Figure12: Meta regression of CRS


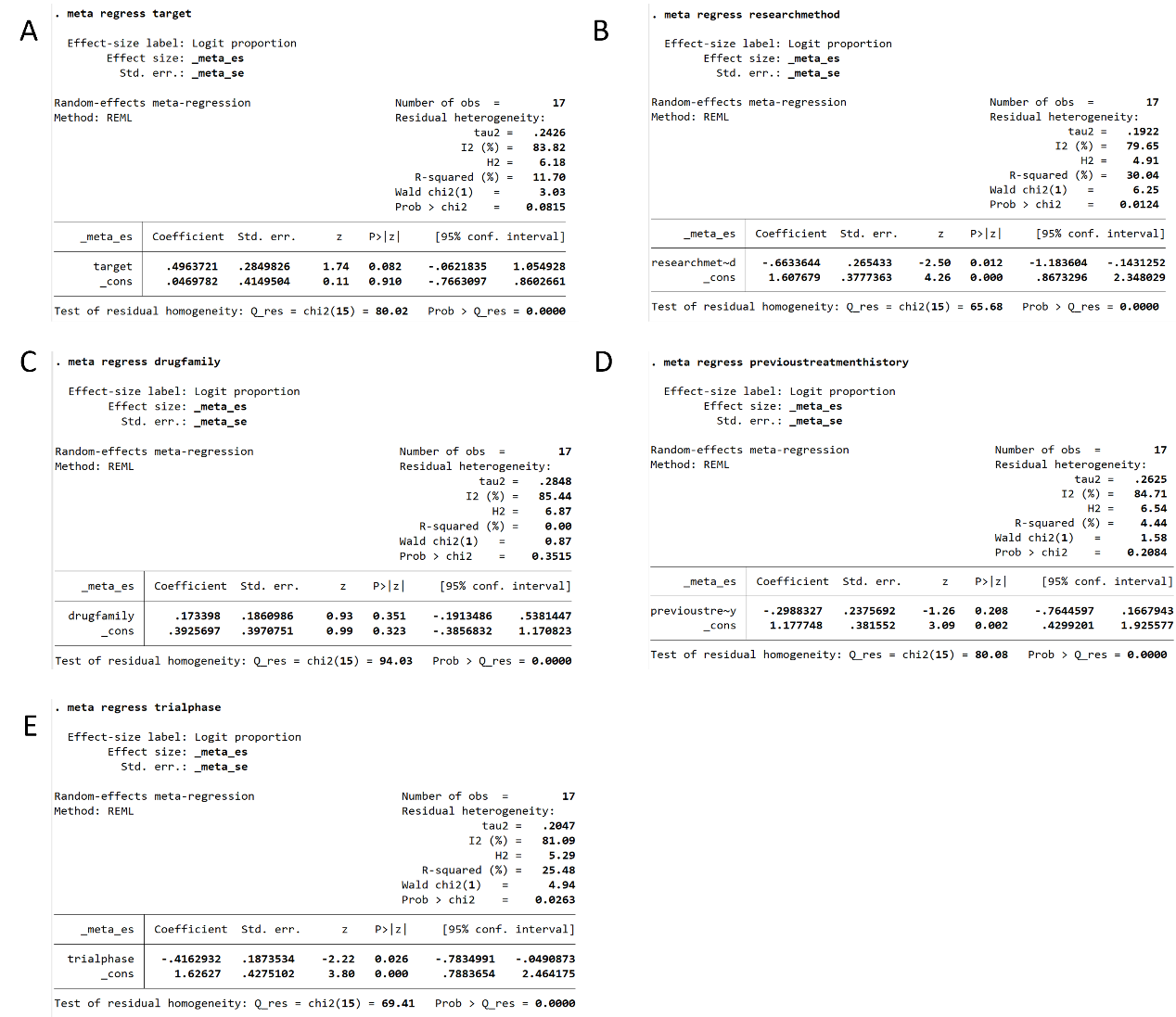


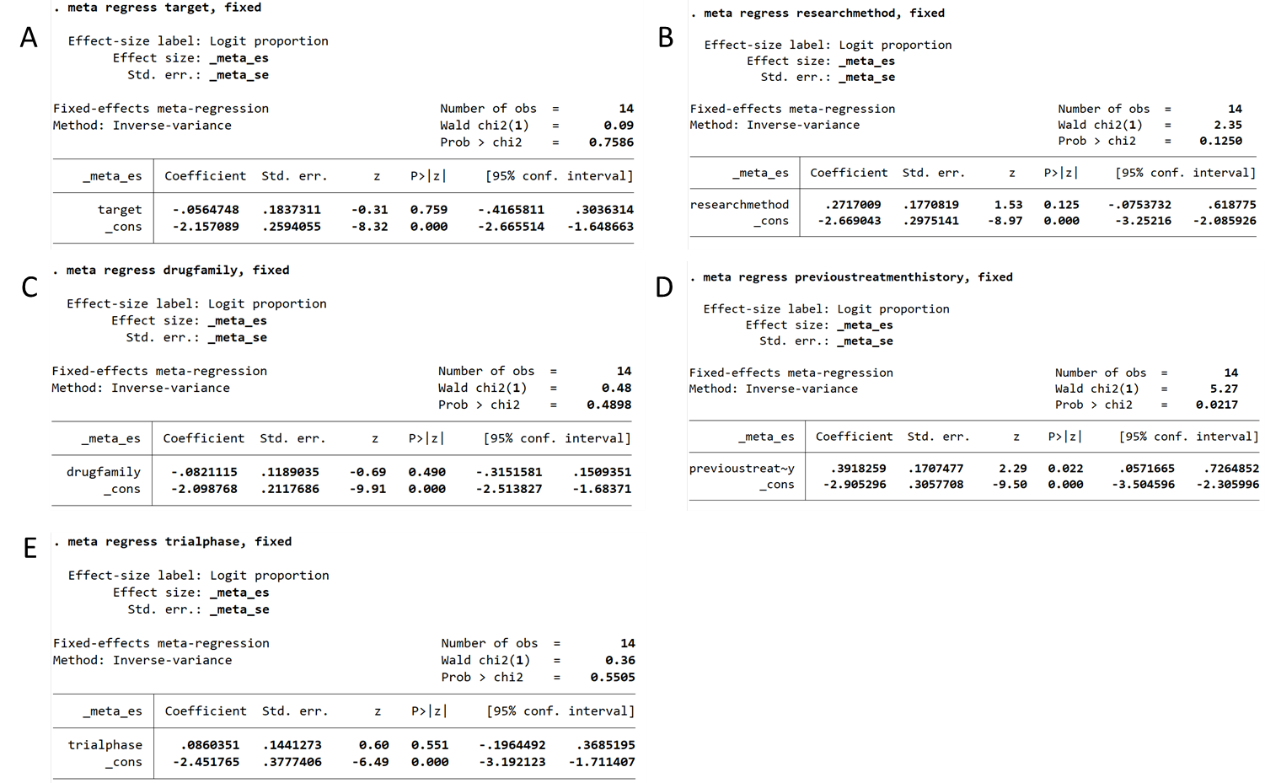


Figure13: Meta regression of ICANS.


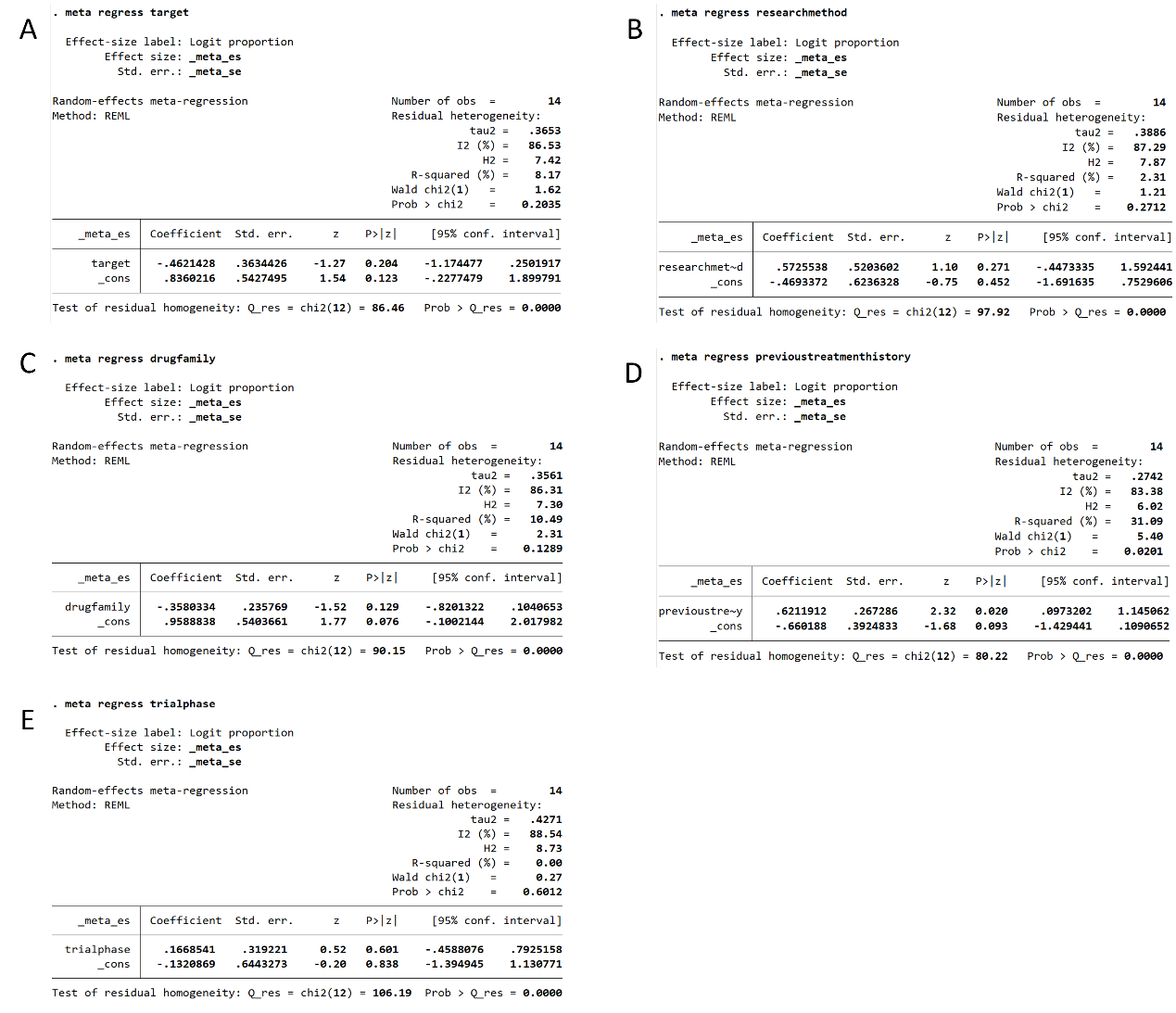


Figure14: Meta regression of Neutropenia.


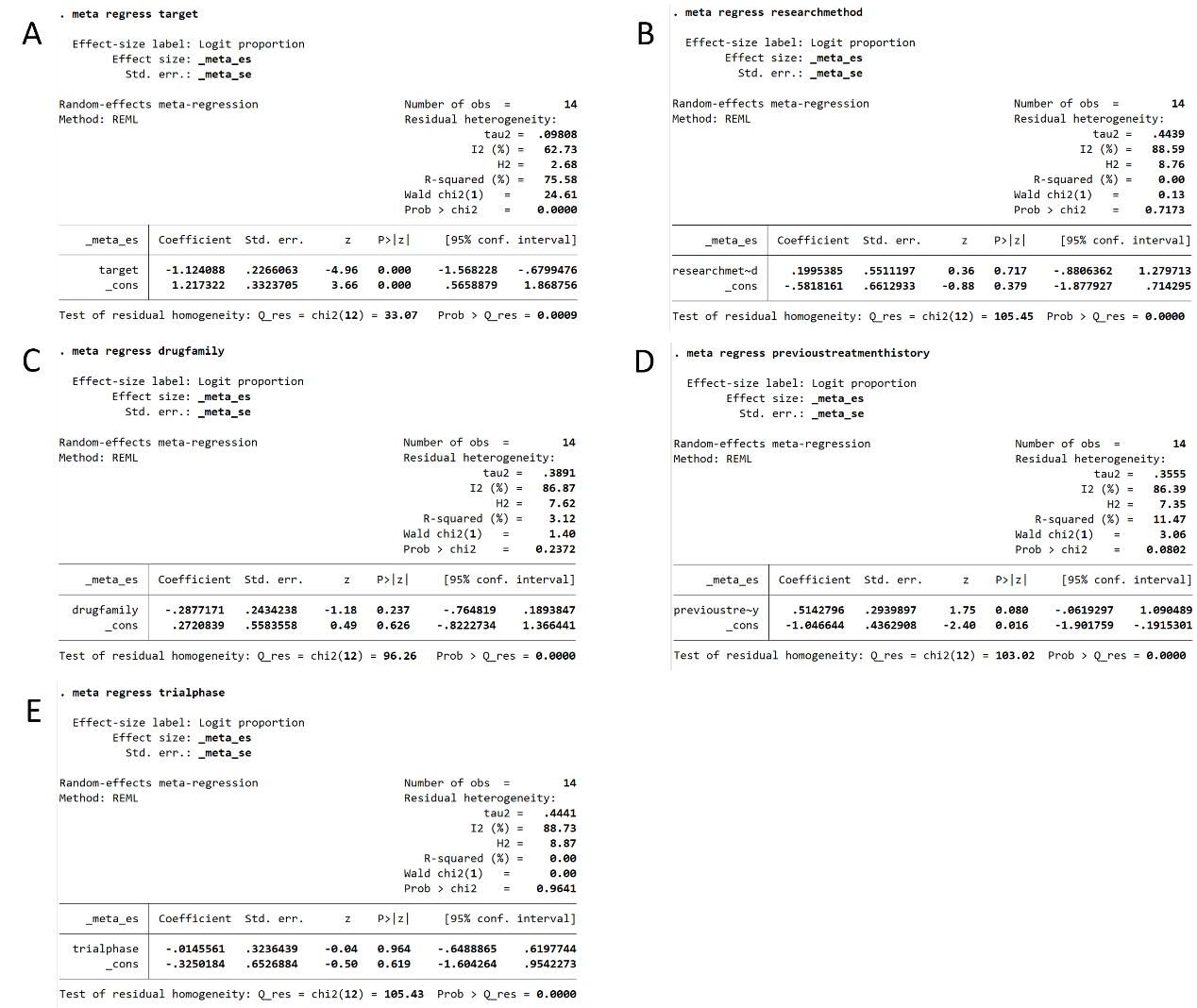


Figure15: Meta regression of grade ≥3 Neutropenia.


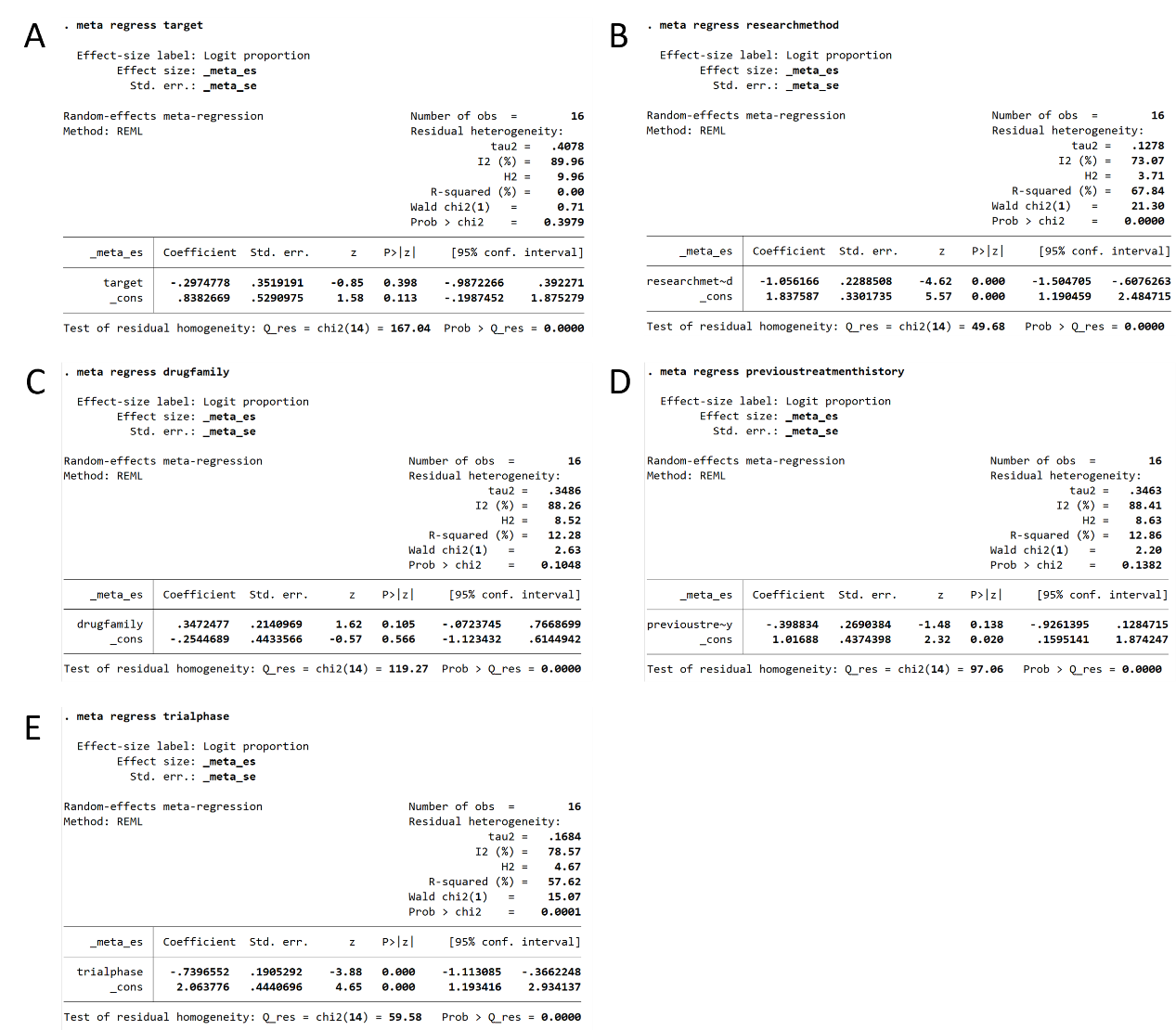


Figure16: Meta regression of infection.


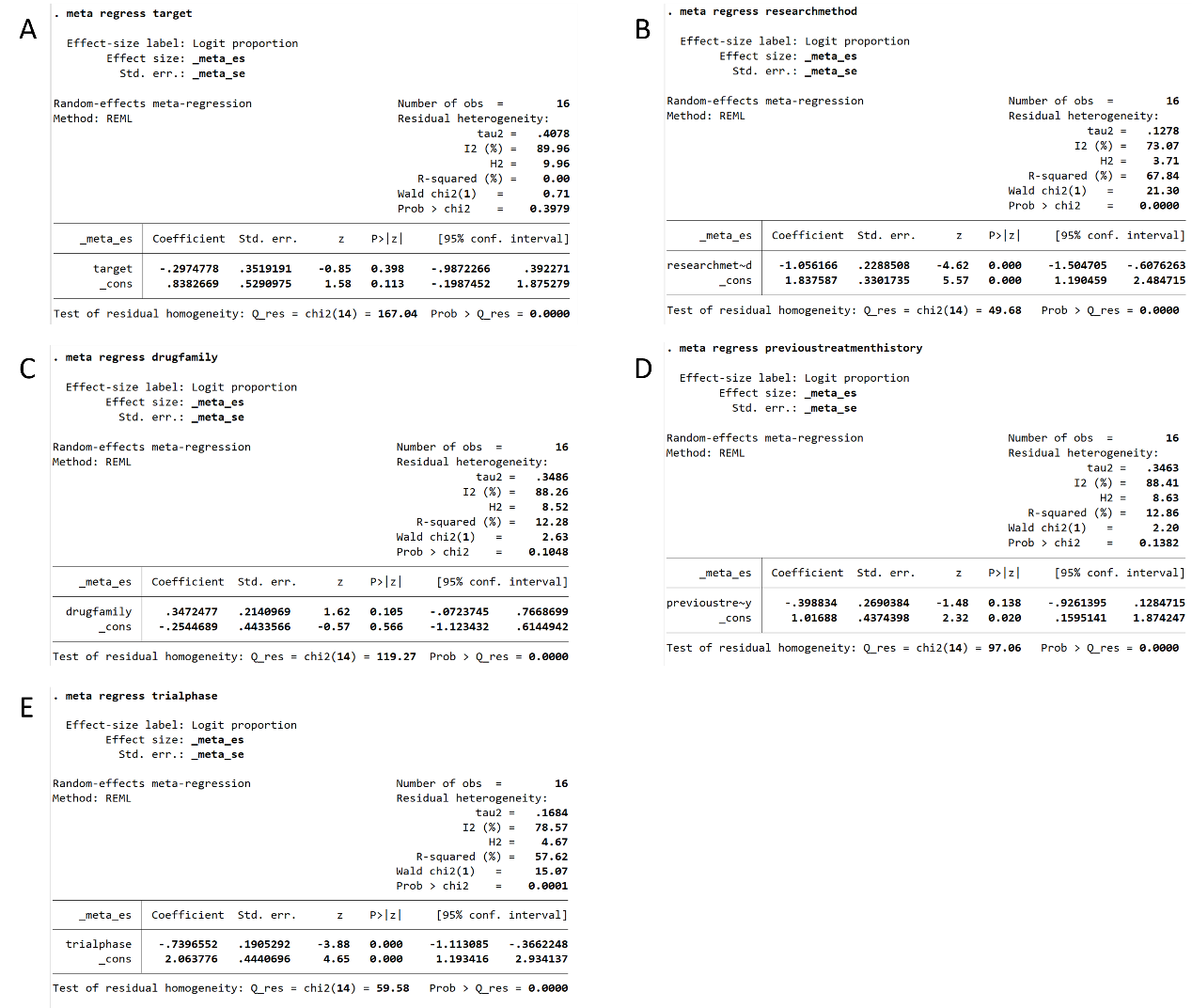


Figure17: Meta regression of grade ≥3 infection.
