# Supplementary material for: Efficacy and safety of BCMA- or GPRC5D-directed CD3 bispecific antibodies in relapsed/refractory multiple myeloma: a systematic review and meta-analysis of prospective clinical trials and real-world studies
Source: Front Immunol. 2026 May 20;17:1811816. doi: 10.3389/fimmu.2026.1811816 (PMC13230190; doi:10.3389/fimmu.2026.1811816)
Supplement: Supplementary file 1 [file DataSheet1.zip › Supplementary File6 Subgroup analysis of efficacy.docx]

***Supplementary File6 Subgroup analysis of efficacy***


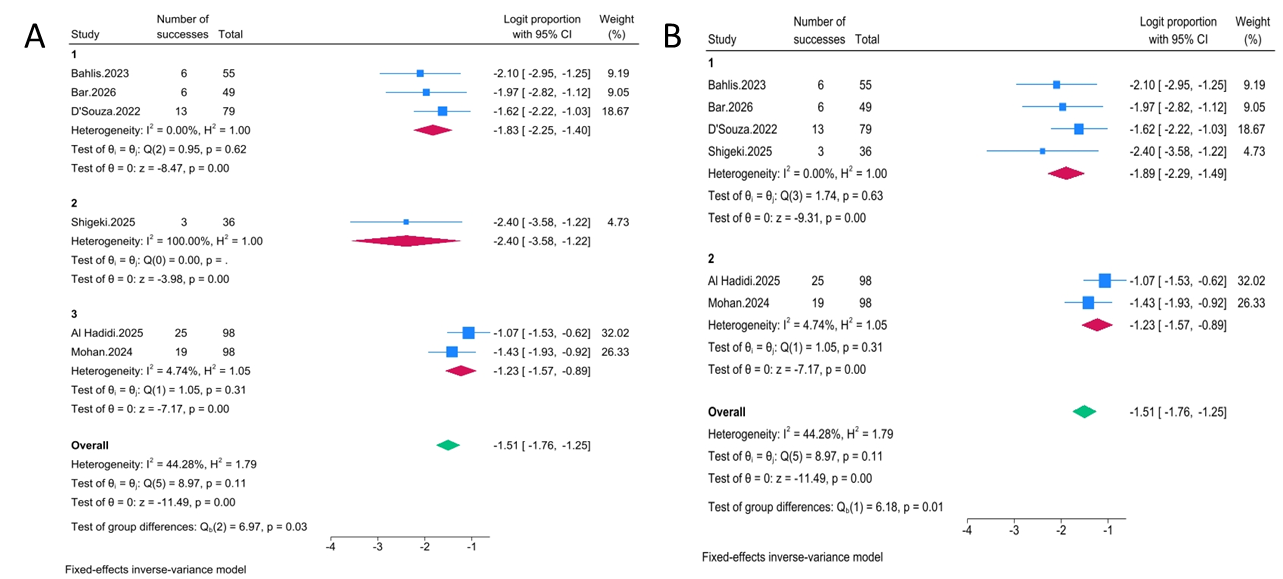


Figure18: CR subgroup analysis. A: Stratified by trial phase, B: Stratified by research method


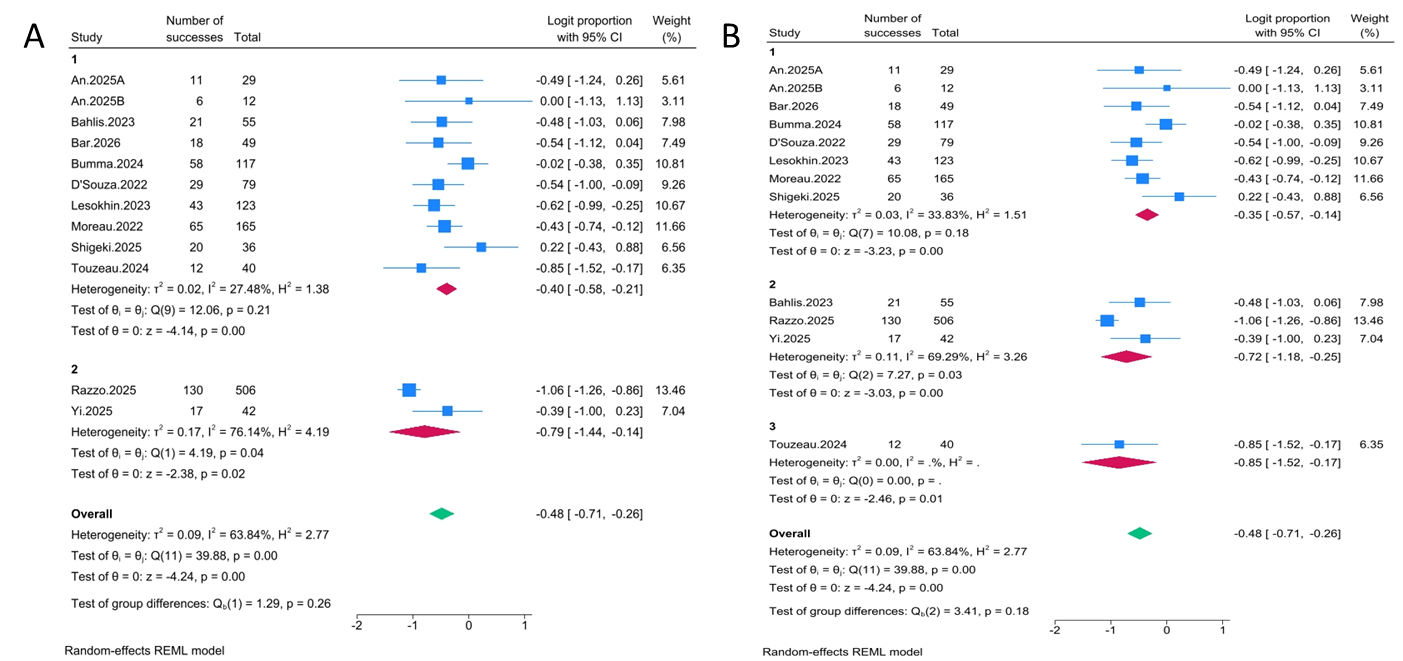


Figure19: ≥CR subgroup analysis A: Stratified by research method, B: Stratified by previous treatment history.


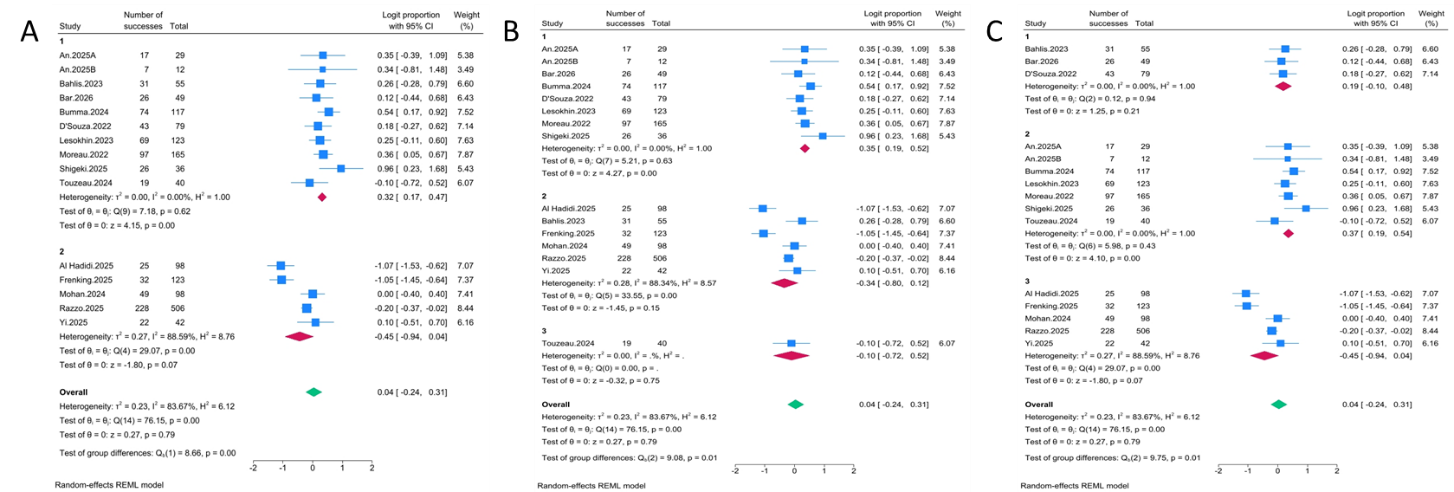


Figure20: ≥VGPR subgroup analysis. A: Stratified by research method, B: Stratified by previous treatment history, C: Stratified by trial phase.


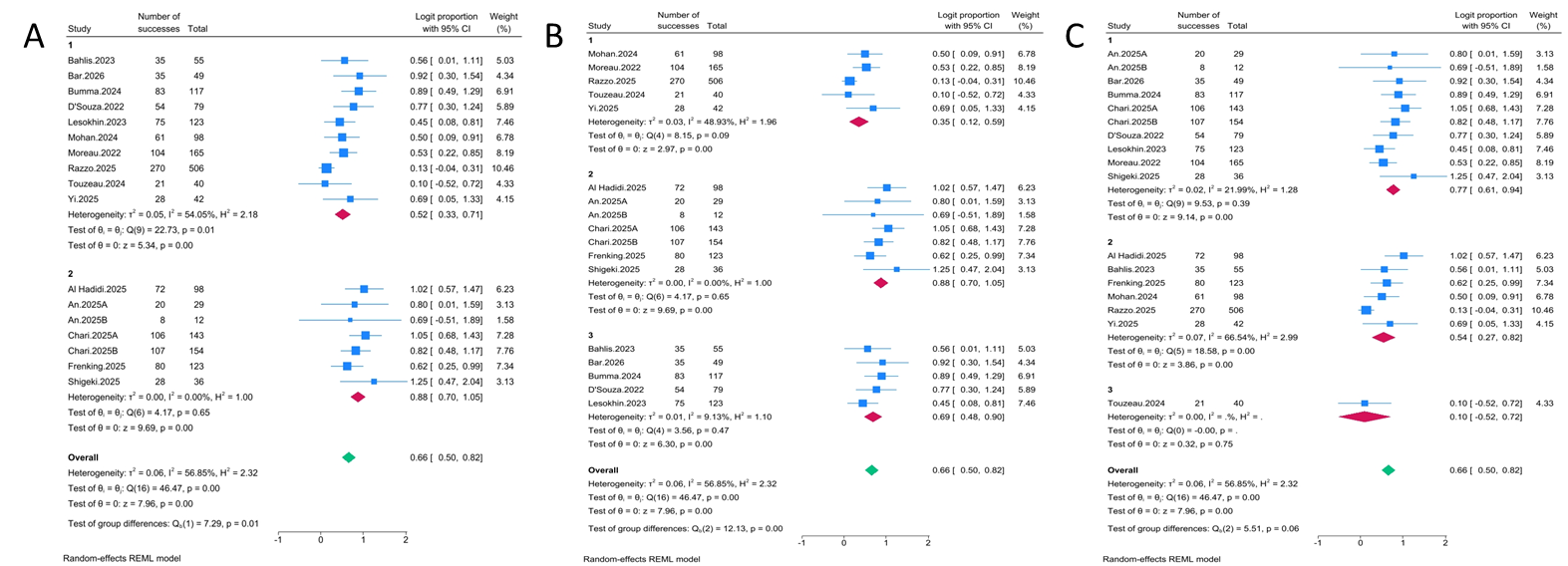


Figure21: ORR subgroup analysis. A: Stratified by drug target. B: Stratified by drug family, C: Stratified by previous treatment history
