# Supplementary material for: Efficacy and safety of BCMA- or GPRC5D-directed CD3 bispecific antibodies in relapsed/refractory multiple myeloma: a systematic review and meta-analysis of prospective clinical trials and real-world studies
Source: Front Immunol. 2026 May 20;17:1811816. doi: 10.3389/fimmu.2026.1811816 (PMC13230190; doi:10.3389/fimmu.2026.1811816)
Supplement: Supplementary file 1 [file DataSheet1.zip › Supplementary File7 Subgroup analysis of safety.docx]

***Supplementary File7 Subgroup analysis of safety***


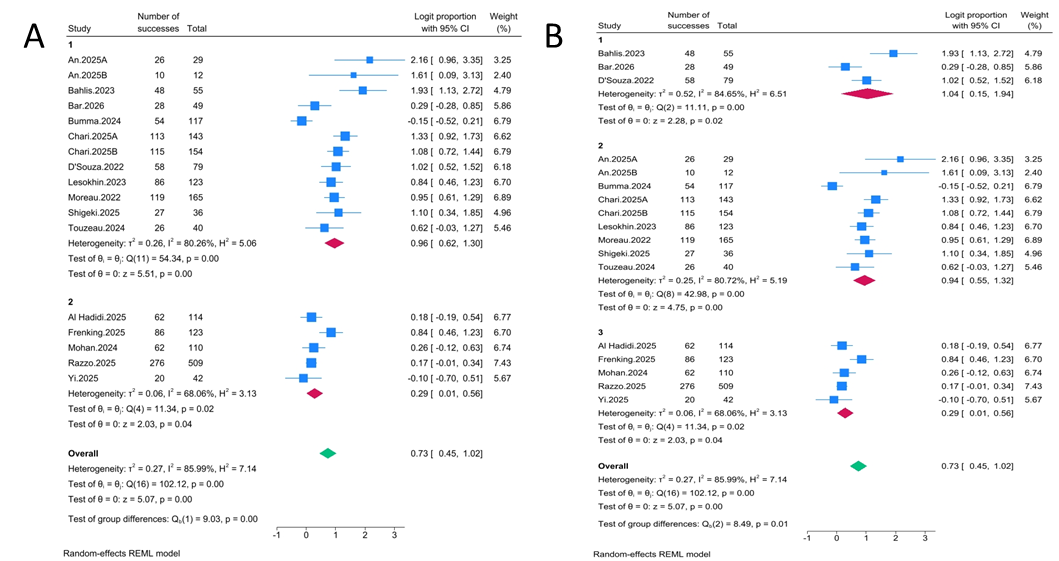


Figure22: CRS subgroup analysis. A: Stratified by research method, B: Stratified by trial phase.


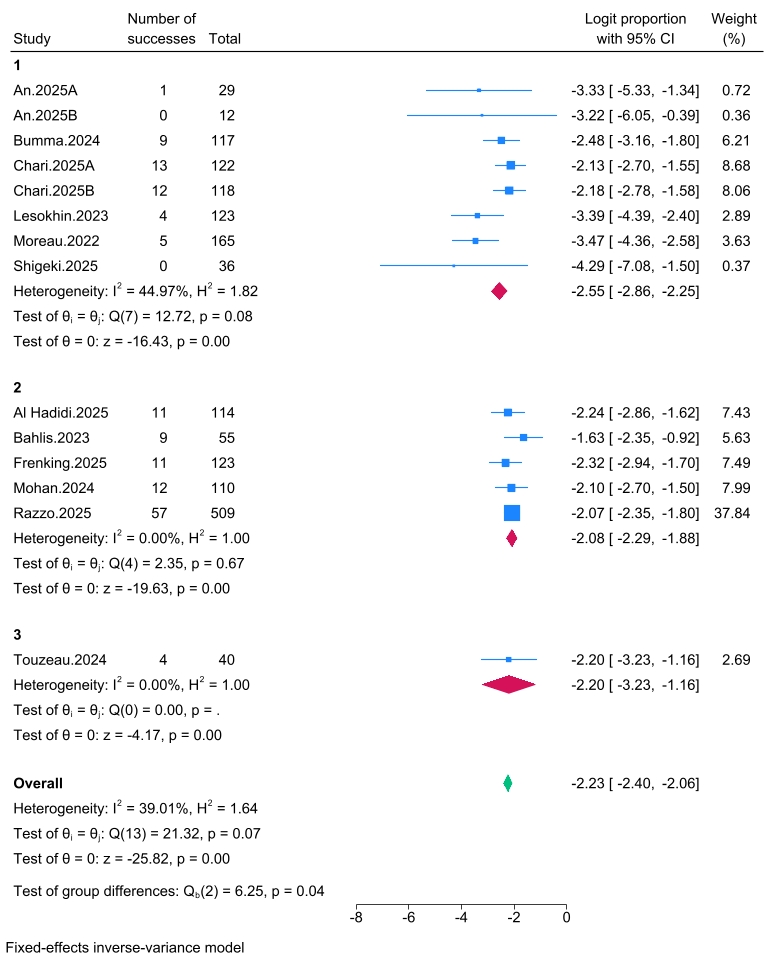


Figure23: Subgroup analysis of ICANS based on previous treatment


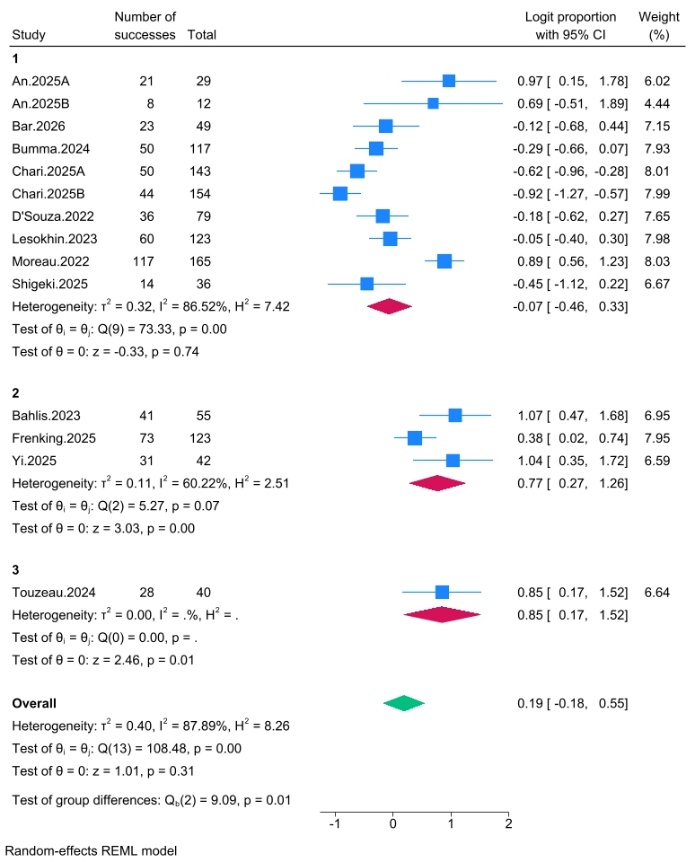


Figure24: Subgroup analysis of neutropenia based on previous treatment


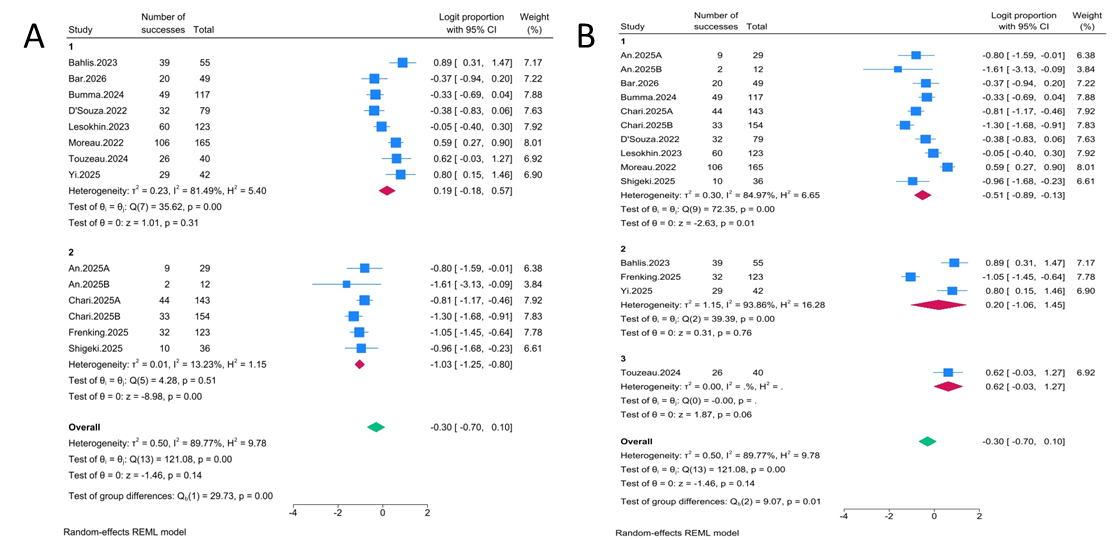


Figure25: Grade ≥3 neutropenia Subgroup analysis. A: Stratified by drug target, B: Stratified by previous treatment.


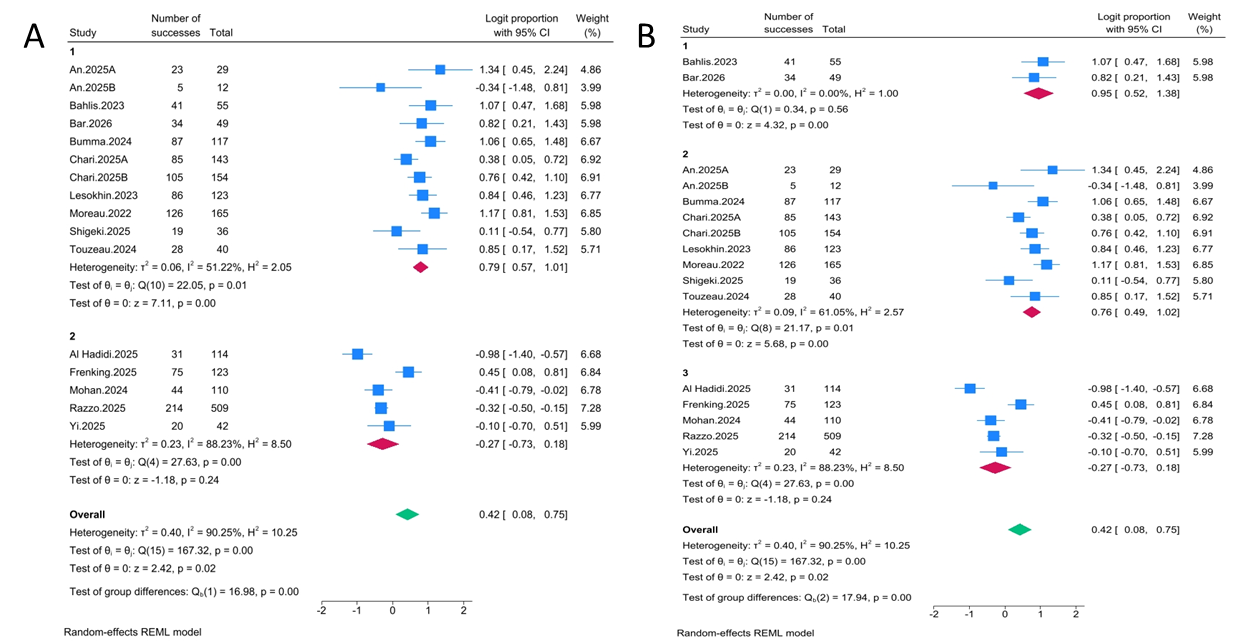


Figure26: Infection subgroup analysis. A: Stratified by research method. B: Stratified by trial phase.


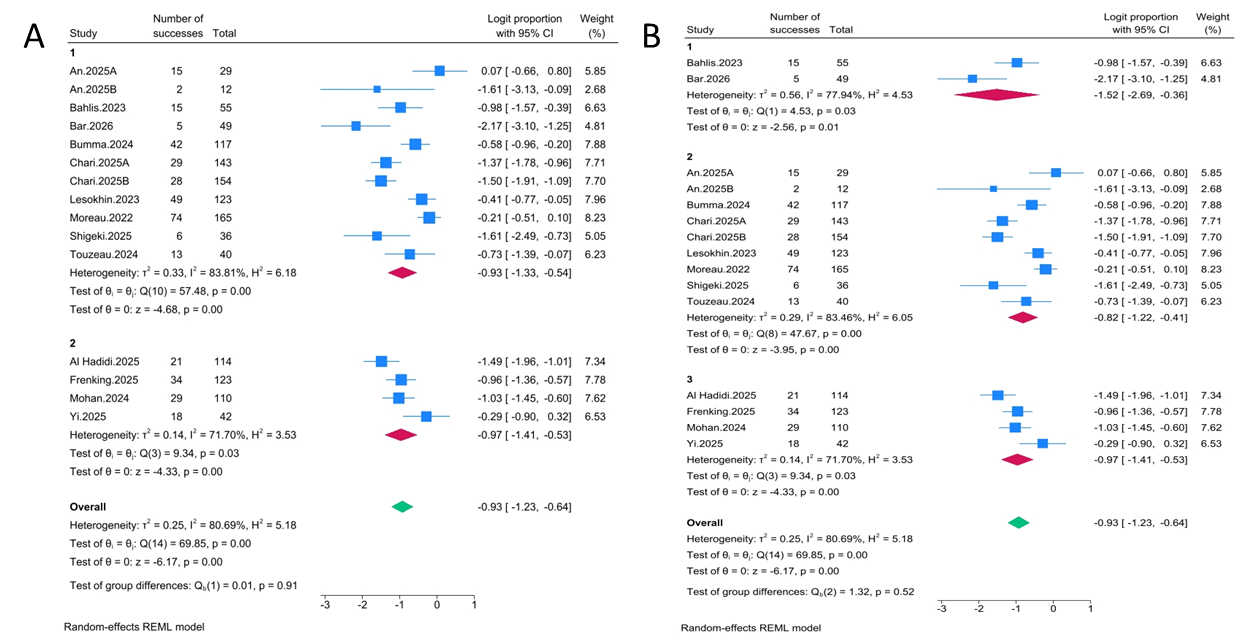


Figure27: Grade ≥3 infection subgroup analysis. A: Stratified by research method. B: Stratified by trial phase.
