# Supplementary material for: Efficacy and safety of BCMA- or GPRC5D-directed CD3 bispecific antibodies in relapsed/refractory multiple myeloma: a systematic review and meta-analysis of prospective clinical trials and real-world studies
Source: Front Immunol. 2026 May 20;17:1811816. doi: 10.3389/fimmu.2026.1811816 (PMC13230190; doi:10.3389/fimmu.2026.1811816)
Supplement: Supplementary file 1 [file DataSheet1.zip › Supplementary File8 Publication bias analysis.docx]

***Publication bias analysis***

Funnel plots were generated for all prespecified outcomes. Formal Egger’s and Begg’s tests were primarily reported for ORR because it was the main efficacy endpoint with a sufficient number of cohorts; funnel plots for outcomes with fewer cohorts should be interpreted cautiously.


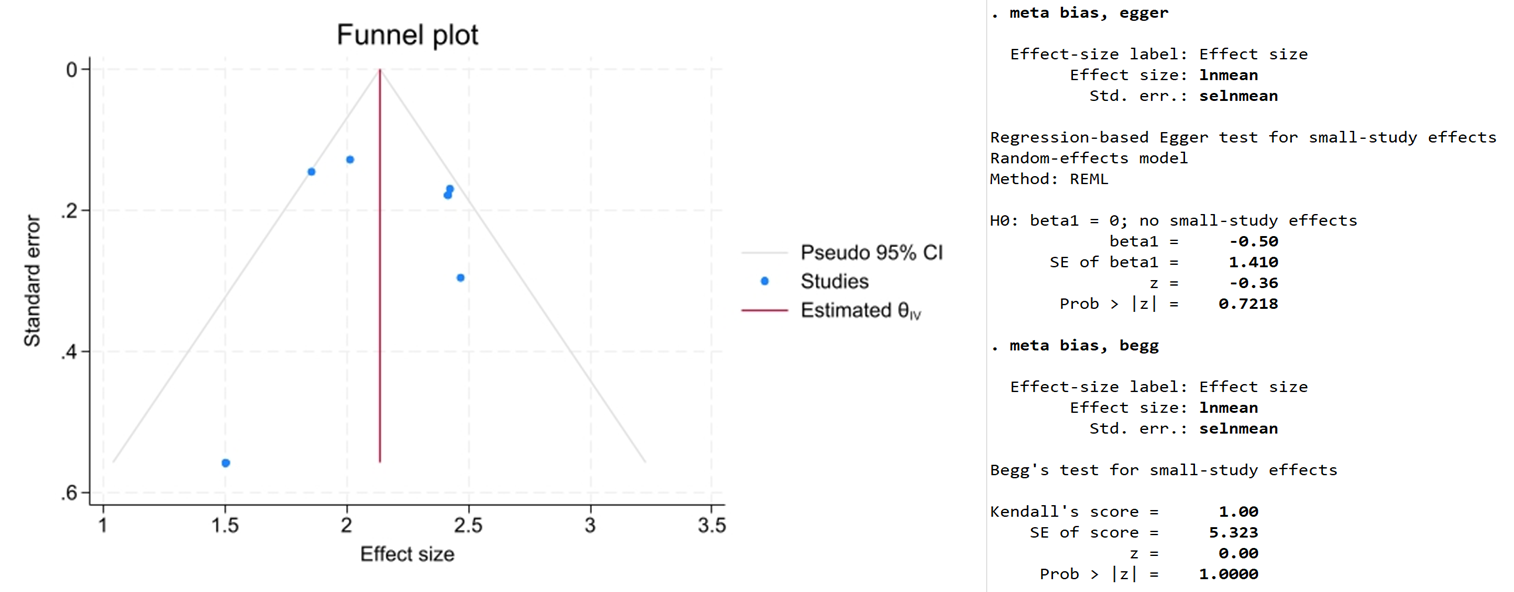


Figure1: Funnel plot for publication bias assessment of PFS


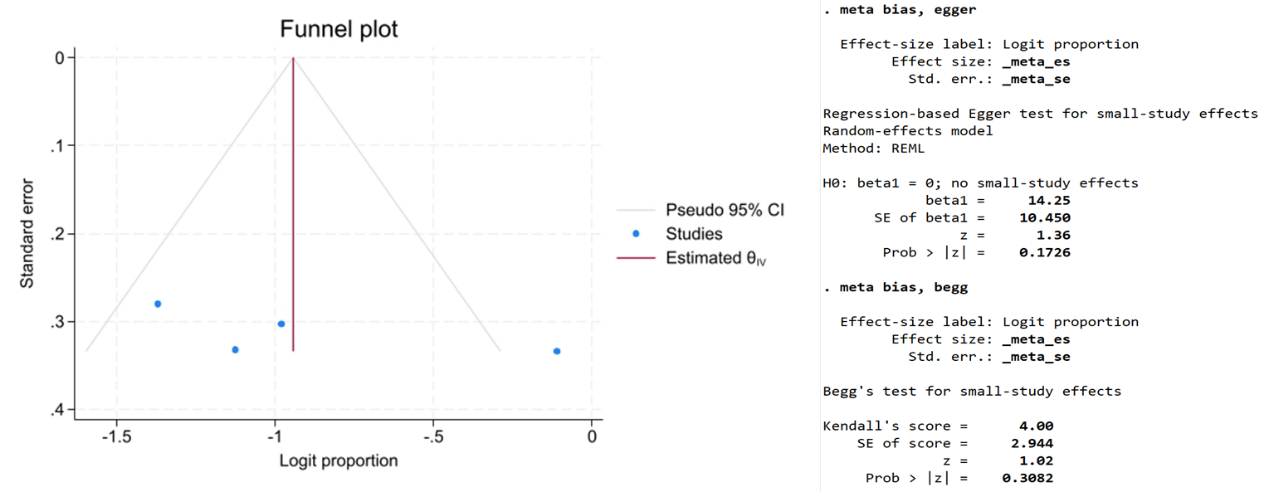


Figure2: Funnel plot for publication bias assessment of sCR.


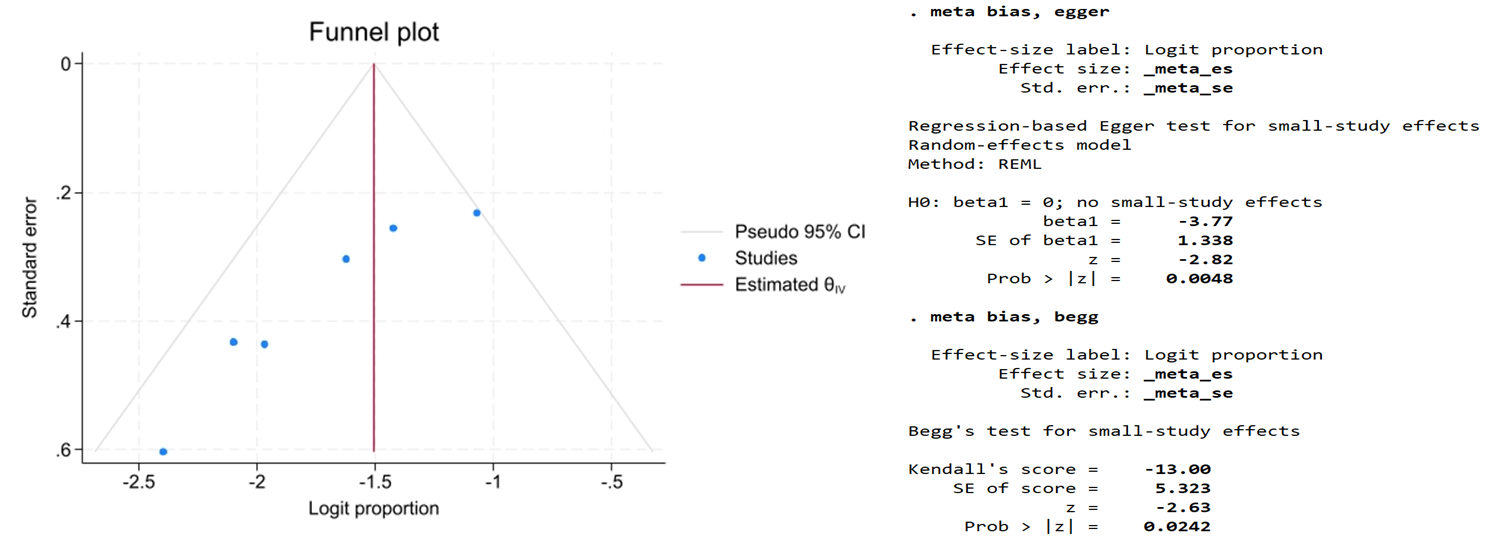


Figure3: Funnel plot for publication bias assessment of CR.


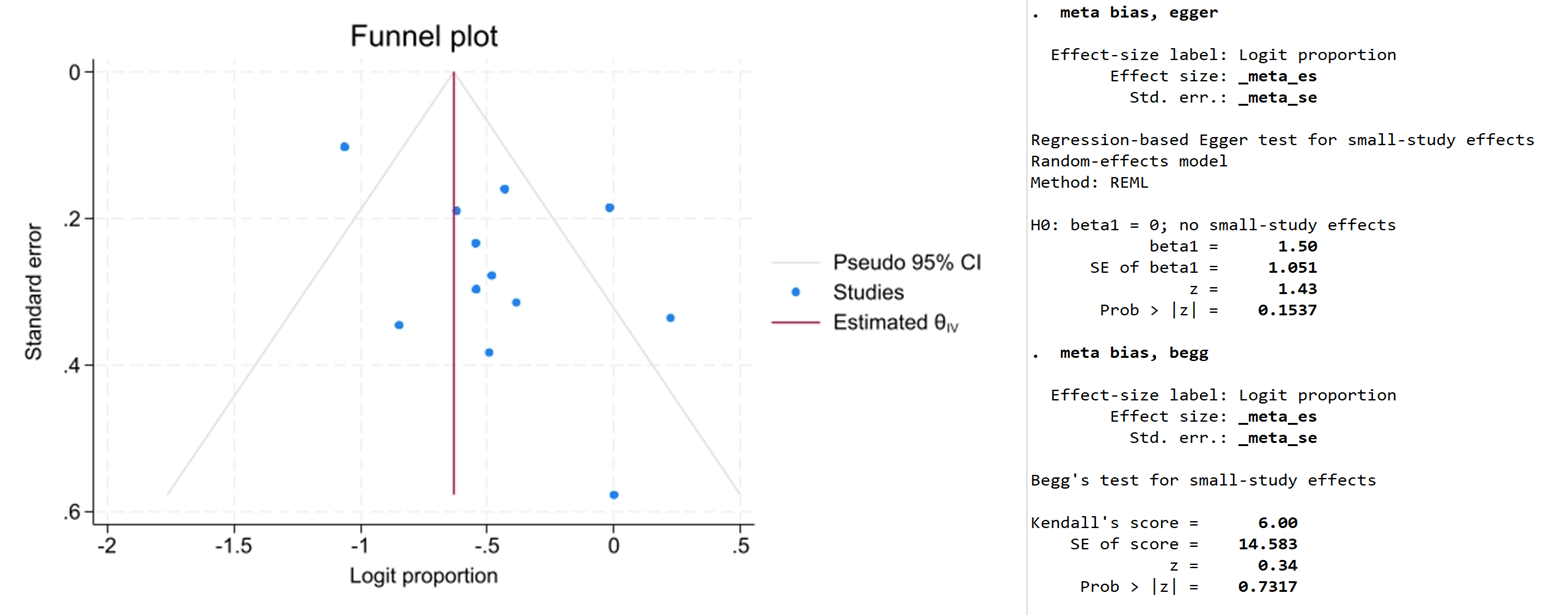


Figure4: Funnel plot for publication bias assessment of ≥CR.


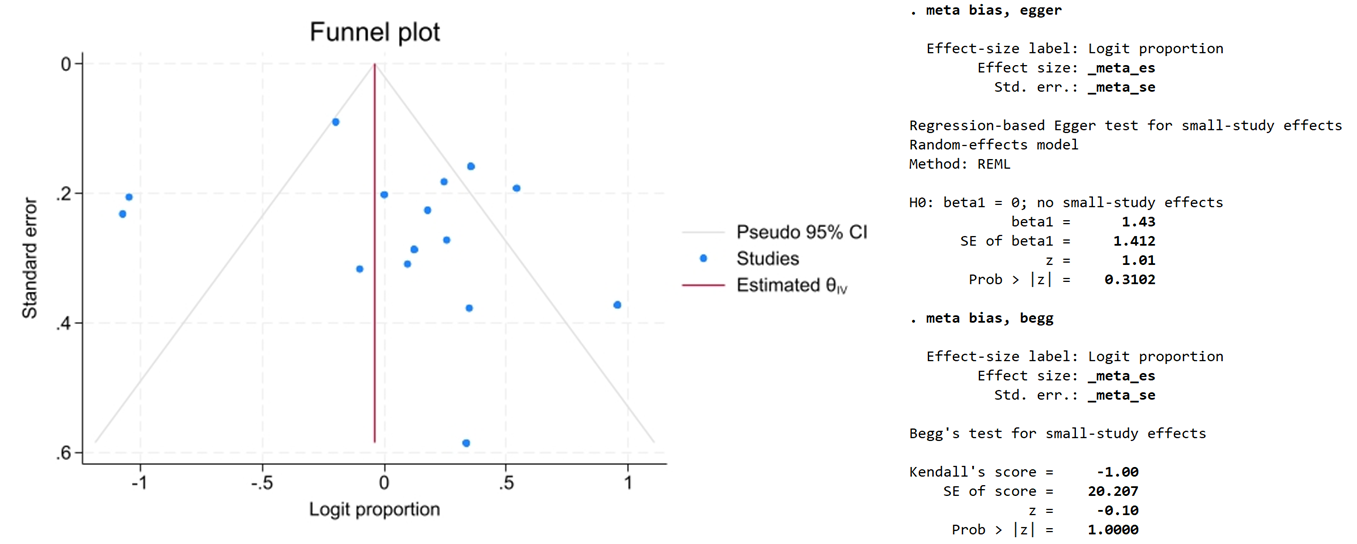


Figure5: Funnel plot for publication bias assessment of ≥VGPR.


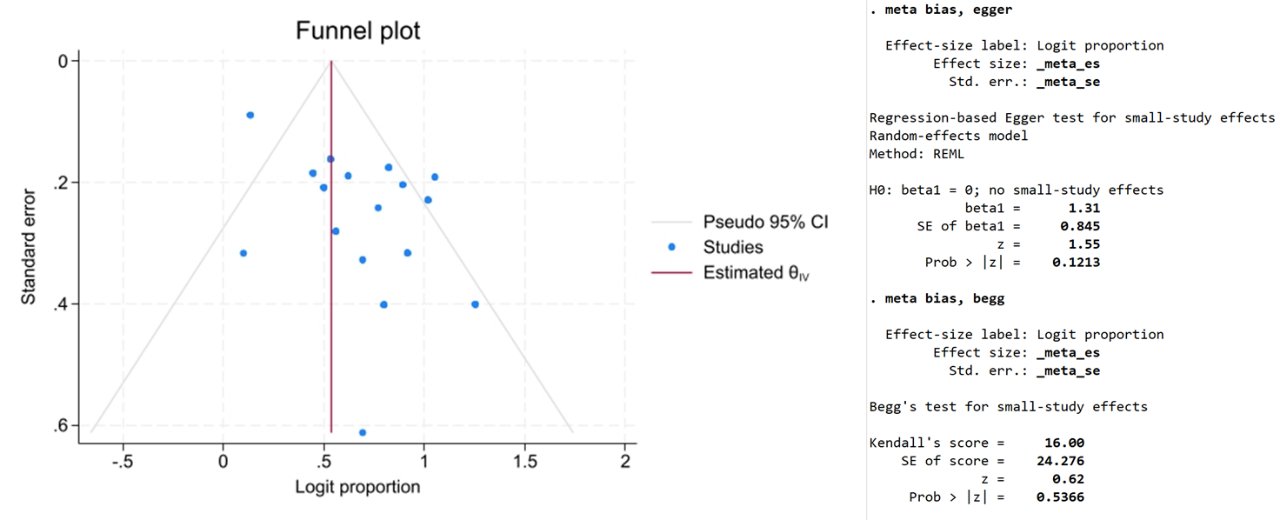


Figure6: Funnel plot for publication bias assessment of ORR.


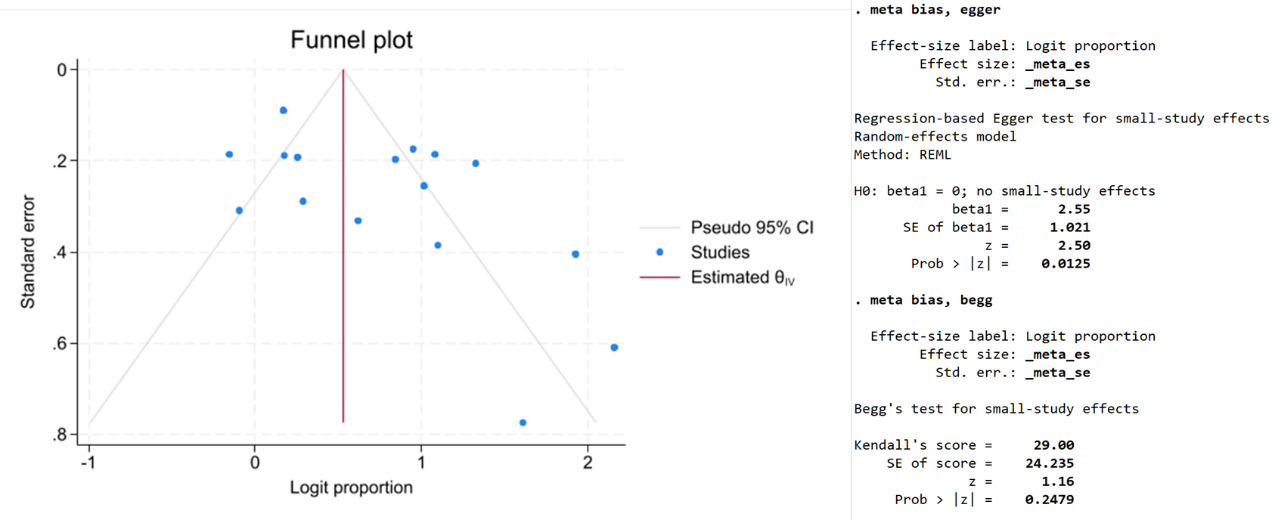


Figure7: Funnel plot for publication bias assessment of CRS.


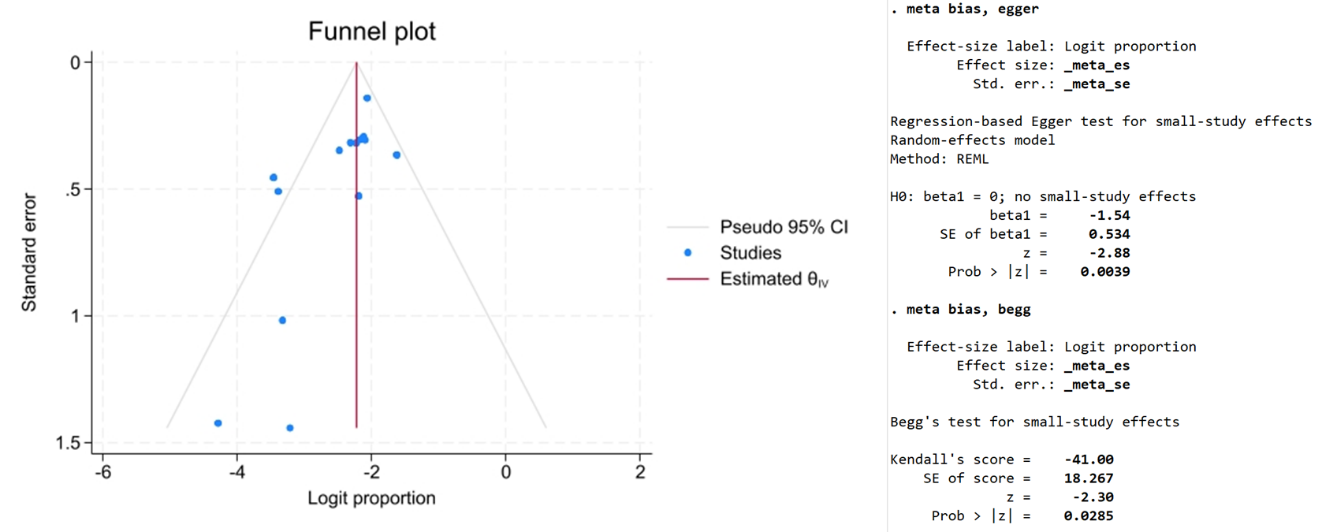


Figure8: Funnel plot for publication bias assessment of ICANS.


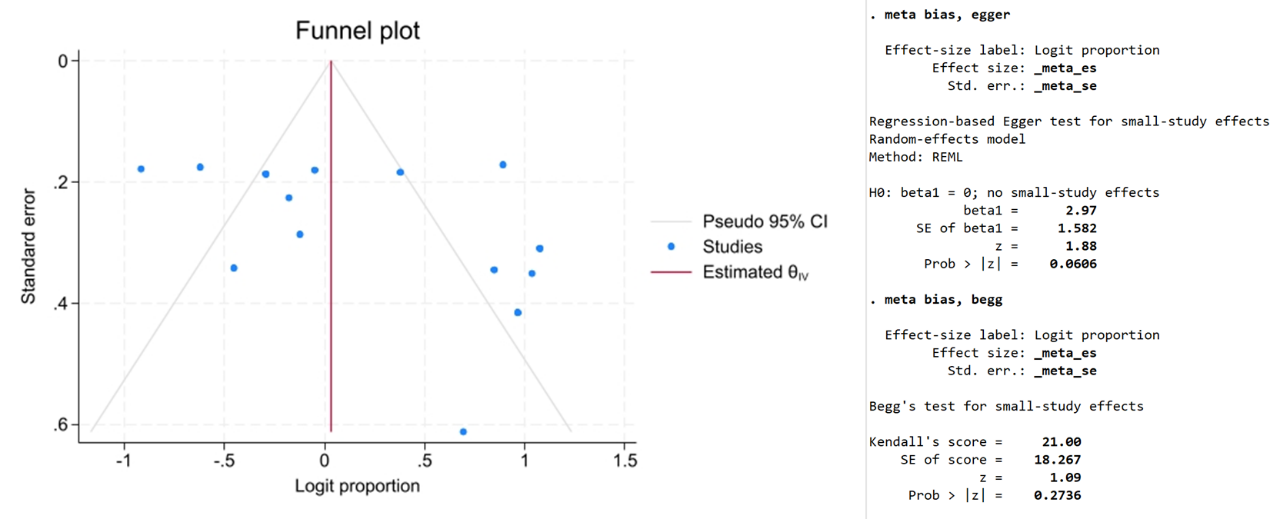


Figure9: Funnel plot for publication bias assessment of Neutropenia.


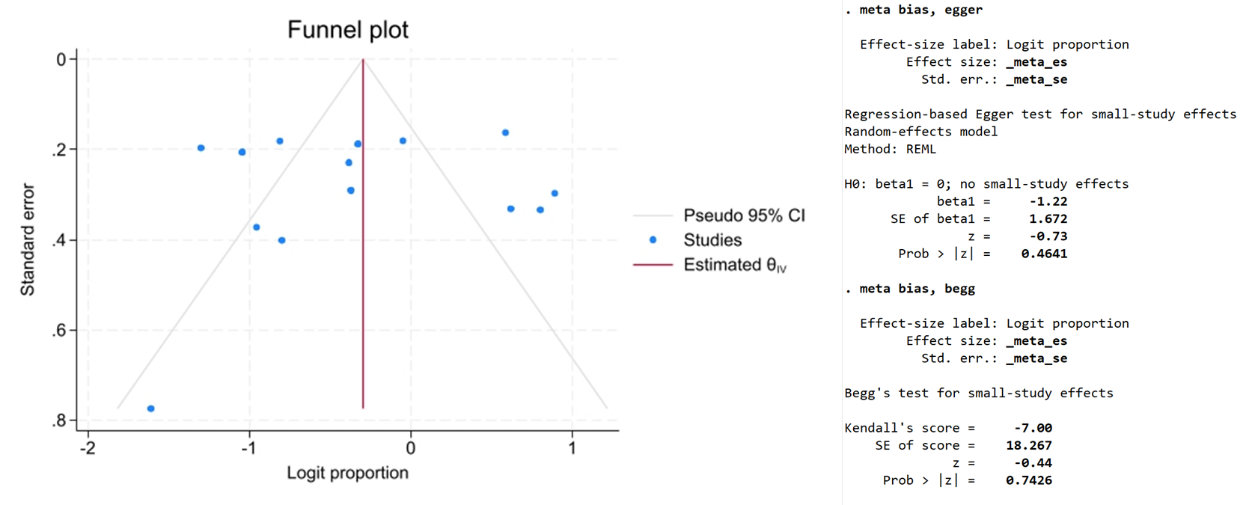


Figure10: Funnel plot for publication bias assessment of Grade ≥3 Neutropenia.


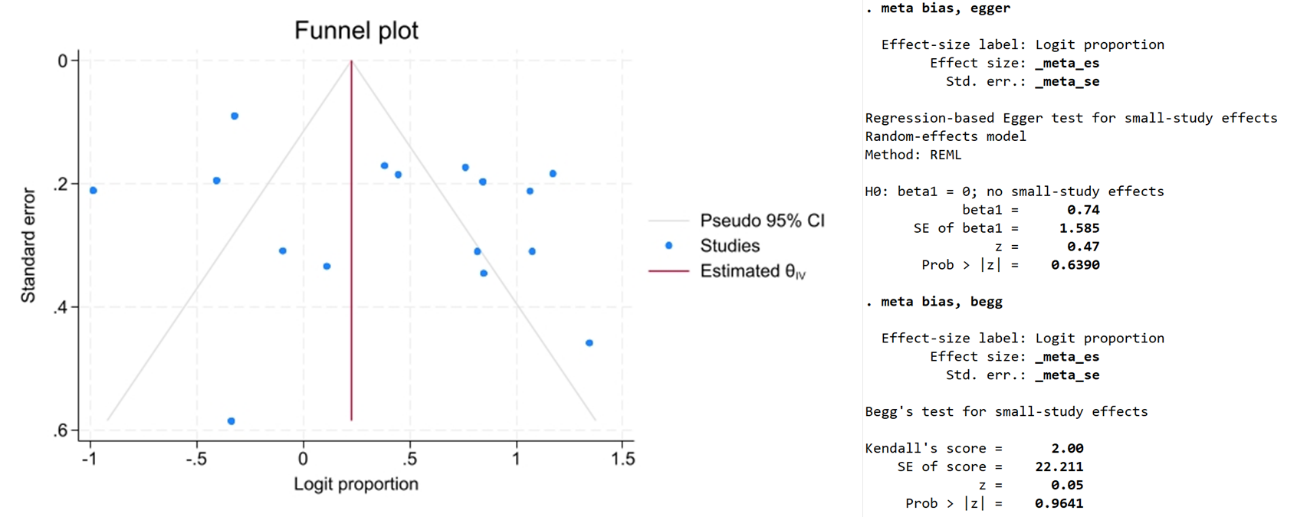


Figure11: Funnel plot for publication bias assessment of Infection.


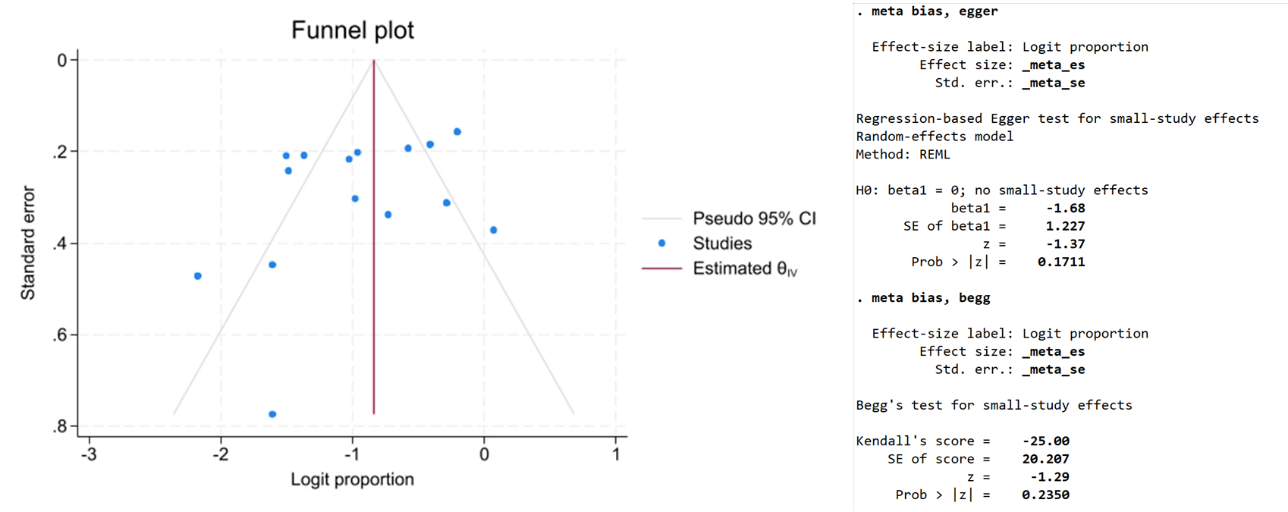


Figure12: Funnel plot for publication bias assessment of Grade ≥3 Infection.


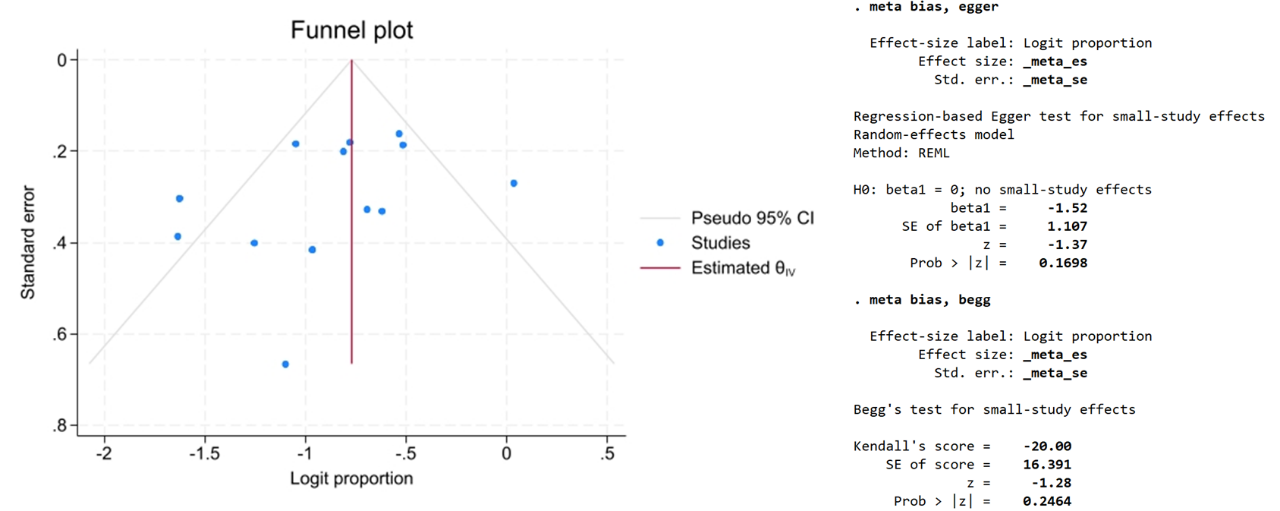


Figure13: Funnel plot for publication bias assessment of Grade ≥3 Anemia.


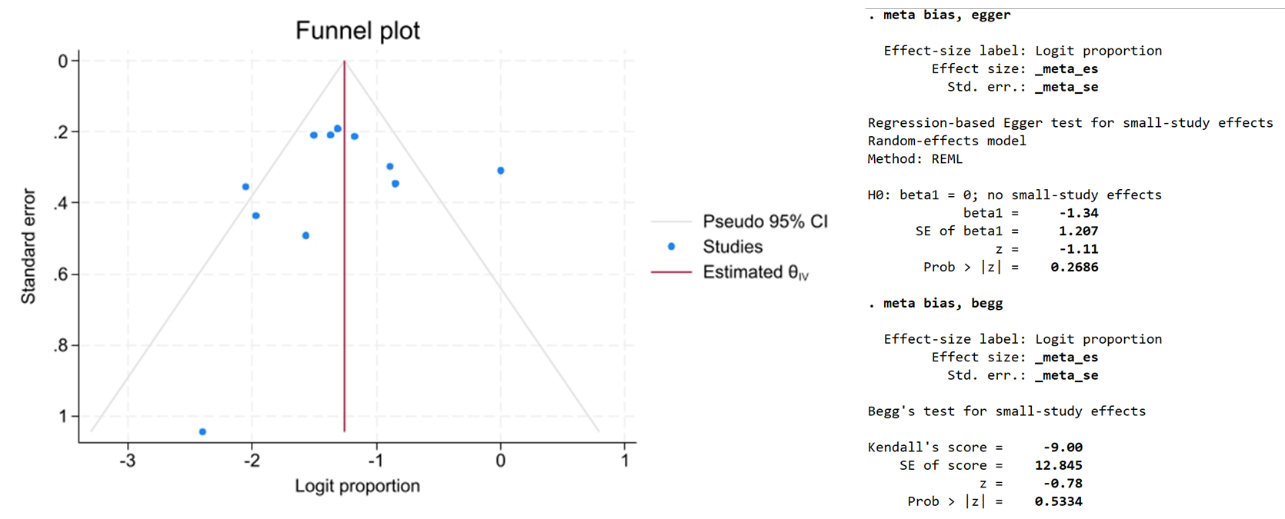


Figure14: Funnel plot for publication bias assessment of Grade ≥3 Thrombocytopenia.
