# Supplementary material for: Efficacy and safety of BCMA- or GPRC5D-directed CD3 bispecific antibodies in relapsed/refractory multiple myeloma: a systematic review and meta-analysis of prospective clinical trials and real-world studies
Source: Front Immunol. 2026 May 20;17:1811816. doi: 10.3389/fimmu.2026.1811816 (PMC13230190; doi:10.3389/fimmu.2026.1811816)
Supplement: Supplementary file 1 [file DataSheet1.zip › Supplementary File1 Search strategy.doc]

**Main Keywords: Bispecific antibody, Multiple Myeloma**

**Pubmed Search strategy**

| #1 | (((((((("Antibodies, Bispecific"[Mesh]) OR (Bispecific antibody[Title/Abstract])) OR (Bifunctional Antibodies[Title/Abstract])) OR (Antibodies, Bifunctional[Title/Abstract])) OR (Bispecific Antibodies[Title/Abstract])) OR (Bispecific Monoclonal Antibodies[Title/Abstract])) OR (Antibodies, Bispecific Monoclonal[Title/Abstract])) OR (Monoclonal Antibodies, Bispecific[Title/Abstract])) |
| --- | --- |
| #2 | (((((((((((((((((((((("Multiple Myeloma"[Mesh]) OR (Multiple Myelomas[Title/Abstract])) OR (Myelomas, Multiple[Title/Abstract])) OR (Myeloma, Plasma-Cell[Title/Abstract])) OR (Myeloma, Plasma Cell[Title/Abstract])) OR (Myelomas, Plasma-Cell[Title/Abstract])) OR (Plasma-Cell Myeloma[Title/Abstract])) OR (Plasma-Cell Myelomas[Title/Abstract])) OR (Myeloma-Multiple[Title/Abstract])) OR (Myeloma Multiple[Title/Abstract])) OR (Myeloma-Multiples[Title/Abstract])) OR (Myeloma, Multiple[Title/Abstract])) OR (Plasma Cell Myeloma[Title/Abstract])) OR (Cell Myeloma, Plasma[Title/Abstract])) OR (Cell Myelomas, Plasma[Title/Abstract])) OR (Myelomas, Plasma Cell[Title/Abstract])) OR (Plasma Cell Myelomas[Title/Abstract])) OR (Kahler Disease[Title/Abstract])) OR (Disease, Kahler[Title/Abstract])) OR (Myelomatosis[Title/Abstract])) OR (Myelomatoses[Title/Abstract])) OR (MM[Title/Abstract])) |
| #3 | #1 AND #2 |

**Embase Search Strategy**

| #1 | 'bispecific antibody'/exp OR 'bispecific antibody' |
| --- | --- |
| #2 | 'bispecific antibody'/exp OR 'bispecific antibody' OR (bispecific AND ('antibody'/exp OR antibody)) OR 'bifunctional antibodies':ti,ab,kw OR 'antibodies, bifunctional':ti,ab,kw OR 'bispecific antibodies':ti,ab,kw OR 'bispecific monoclonal antibodies':ti,ab,kw OR 'antibodies, bispecific monoclonal':ti,ab,kw OR 'monoclonal antibodies, bispecific':ti,ab,kw OR 'antibodies, bispecific':ti,ab,kw OR 'bispecific immunoglobulin':ti,ab,kw OR 'immunoglobulin, bispecific':ti,ab,kw |
| #3 | #1 AND #2 |
| #4 | 'multiple myeloma'/exp OR 'multiple myeloma' |
| #5 | 'multiple myeloma'/exp OR 'multiple myeloma' OR (multiple AND ('myeloma'/exp OR myeloma)) OR 'multiple myelomas':ti,ab,kw OR 'myelomas, multiple':ti,ab,kw OR 'myeloma, plasma-cell':ti,ab,kw OR 'myeloma, plasma cell':ti,ab,kw OR 'myelomas, plasma-cell':ti,ab,kw OR 'plasma-cell myeloma':ti,ab,kw OR 'plasma-cell myelomas':ti,ab,kw OR 'myeloma multiple':ti,ab,kw OR 'myeloma multiples':ti,ab,kw OR 'myeloma, multiple':ti,ab,kw OR 'plasma cell myeloma':ti,ab,kw OR 'cell myeloma, plasma':ti,ab,kw OR 'cell myelomas, plasma':ti,ab,kw OR 'myelomas, plasma cell':ti,ab,kw OR 'plasma cell myelomas':ti,ab,kw OR 'kahler disease':ti,ab,kw OR 'morbus kahler':ti,ab,kw OR 'myeloma multiplex':ti,ab,kw OR 'disease, kahler':ti,ab,kw OR myelomatosis:ti,ab,kw OR myelomatoses:ti,ab,kw |
| #6 | #4 OR #5 |
| #7 | #3 AND #6 |

**Web of science search strategy**

| #1 | Bispecific antibody (Topic) or Bifunctional Antibodies (Topic) or Antibodies, Bifunctional (Topic) or Bispecific Antibodies (Topic) or Bispecific Monoclonal Antibodies (Topic) or Antibodies, Bispecific Monoclonal (Topic) or Monoclonal Antibodies, Bispecific (Topic) or antibodies, bispecific (Topic) or bispecific immunoglobulin (Topic) or immunoglobulin, bispecific (Topic) |
| --- | --- |
| #2 | multiple myeloma (Topic) or Multiple Myelomas (Topic) or myeloma, multiple (Topic) or Myelomas, Multiple (Topic) or Myeloma, Plasma-Cell (Topic) or Myeloma, Plasma Cell (Topic) or Myelomas, Plasma-Cell (Topic) or Plasma-Cell Myeloma (Topic) or Plasma-Cell Myelomas (Topic) or Myeloma-Multiple (Topic) or Myeloma Multiple (Topic) or Myeloma-Multiples (Topic) or Myeloma, Multiple (Topic) or Plasma Cell Myeloma (Topic) or Cell Myeloma, Plasma (Topic) or Cell Myelomas, Plasma (Topic) or Myelomas, Plasma Cell (Topic) or Plasma Cell Myelomas (Topic) or Kahler Disease (Topic) or morbus kahler (Topic) or myeloma multiplex (Topic) or Disease, Kahler (Topic) or Myelomatosis (Topic) or Myelomatoses (Topic) |
| #3 | #1 AND #2 |

**Cochrane Library search strategy**

| #1 | MeSH descriptor: [Antibodies, Bispecific] explode all trees |
| --- | --- |
| #2 | (Bispecific antibody):ti,ab,kw OR (Bifunctional Antibodies):ti,ab,kw OR (Antibodies, Bifunctional):ti,ab,kw OR (Bispecific Antibodies):ti,ab,kw OR (Bispecific Monoclonal Antibodies):ti,ab,kw |
| #3 | (Antibodies, Bispecific Monoclonal):ti,ab,kw OR (Monoclonal Antibodies, Bispecific):ti,ab,kw OR (antibodies, bispecific):ti,ab,kw OR (bispecific immunoglobulin):ti,ab,kw OR (immunoglobulin, bispecific):ti,ab,kw |
| #4 | #1 OR #2 OR #3 |
| #5 | MeSH descriptor: [Multiple Myeloma] explode all trees |
| #6 | (multiple myeloma):ti,ab,kw OR (Multiple Myelomas):ti,ab,kw OR (myeloma, multiple):ti,ab,kw OR (Myelomas, Multiple):ti,ab,kw OR (Myeloma, Plasma-Cell):ti,ab,kw |
| #7 | (Myeloma, Plasma Cell):ti,ab,kw OR (Myelomas, Plasma-Cell):ti,ab,kw OR (Plasma-Cell Myeloma):ti,ab,kw OR (Plasma-Cell Myelomas):ti,ab,kw OR (Myeloma-Multiple):ti,ab,kw |
| #8 | (Myeloma Multiple):ti,ab,kw OR (Myeloma-Multiples):ti,ab,kw OR (Myeloma, Multiple):ti,ab,kw OR (Plasma Cell Myeloma):ti,ab,kw OR (Cell Myeloma, Plasma):ti,ab,kw |
| #9 | (Cell Myelomas, Plasma):ti,ab,kw OR (Myelomas, Plasma Cell):ti,ab,kw OR (Plasma Cell Myelomas):ti,ab,kw OR (Kahler Disease):ti,ab,kw OR (morbus kahler):ti,ab,kw |
| #10 | (myeloma multiplex):ti,ab,kw OR (Disease, Kahler):ti,ab,kw OR (Myelomatosis):ti,ab,kw OR (Myelomatoses):ti,ab,kw |
| #11 | #5 OR #6 OR #7 OR #8 OR #9 OR #10 |
| #12 | #4 AND #11 |

**Scopus search strategy**

| #1 | ( TITLE-ABS-KEY ( Bispecific antibody ) OR TITLE-ABS-KEY ( Bifunctional Antibodies ) OR TITLE-ABS-KEY ( Antibodies , Bifunctional ) OR TITLE-ABS-KEY ( Bispecific Antibodies ) OR TITLE-ABS-KEY ( Bispecific Monoclonal Antibodies ) OR TITLE-ABS-KEY ( Antibodies , Bispecific Monoclonal ) OR TITLE-ABS-KEY ( Monoclonal Antibodies , Bispecific ) OR TITLE-ABS-KEY ( antibodies , bispecific ) OR TITLE-ABS-KEY ( bispecific immunoglobulin ) OR TITLE-ABS-KEY ( immunoglobulin , bispecific ) ) |
| --- | --- |
| #2 | ( TITLE-ABS-KEY ( multiple myeloma ) OR TITLE-ABS-KEY ( Multiple Myelomas ) OR TITLE-ABS-KEY ( myeloma , multiple ) OR TITLE-ABS-KEY ( Myelomas , Multiple ) OR TITLE-ABS-KEY ( Myeloma , Plasma-Cell ) OR TITLE-ABS-KEY ( Myeloma , Plasma Cell ) OR TITLE-ABS-KEY ( Myelomas , Plasma-Cell ) OR TITLE-ABS-KEY ( Plasma-Cell Myeloma ) OR TITLE-ABS-KEY ( Plasma-Cell Myelomas ) OR TITLE-ABS-KEY ( Myeloma-Multiple ) OR TITLE-ABS-KEY ( Myeloma Multiple ) OR TITLE-ABS-KEY ( Myeloma-Multiples ) OR TITLE-ABS-KEY ( Myeloma , Multiple ) OR TITLE-ABS-KEY ( Plasma Cell Myeloma ) OR TITLE-ABS-KEY ( Cell Myeloma , Plasma ) OR TITLE-ABS-KEY ( Cell Myelomas , Plasma ) OR TITLE-ABS-KEY ( Myelomas , Plasma Cell ) OR TITLE-ABS-KEY ( Plasma Cell Myelomas ) OR TITLE-ABS-KEY ( Kahler Disease ) OR TITLE-ABS-KEY ( morbus kahler ) OR TITLE-ABS-KEY ( myeloma multiplex ) OR TITLE-ABS-KEY ( Disease , Kahler ) OR TITLE-ABS-KEY ( Myelomatosis ) OR TITLE-ABS-KEY ( Myelomatoses ) ) |
| #3 | #1 AND #2 |
